# Immune–epithelial–stromal networks define the cellular ecosystem of the small intestine in celiac disease

**DOI:** 10.1038/s41590-025-02146-2

**Published:** 2025-05-06

**Authors:** Michael E. B. FitzPatrick, Agne Antanaviciute, Melanie Dunstan, Karolina Künnapuu, Dominik Trzupek, Nicholas M. Provine, Kyla Dooley, Jia-Yuan Zhang, Sophie L. Irwin, Lucy C. Garner, Jane I. Pernes, Ricardo C. Ferreira, Sarah C. Sasson, Dominik Aschenbrenner, Devika Agarwal, Astor Rodrigues, Lucy Howarth, Oliver Brain, Darren Ruane, Elizabeth Soilleux, Sarah A. Teichmann, Calliope A. Dendrou, Alison Simmons, Holm H. Uhlig, John A. Todd, Paul Klenerman

**Affiliations:** 1 Translational Gastroenterology and Liver Unit, Nuffield Department of Medicine, University of Oxford, John Radcliffe Hospital, Oxford, UK; 2 MRC Translational Immune Discovery Unit, Weatherall Institute of Molecular Medicine, John Radcliffe Hospital, Oxford, UK; 3 MRC WIMM Centre for Computational Biology, Weatherall Institute of Molecular Medicine, John Radcliffe Hospital, Oxford, UK; 4 Centre for Human Genetics, Nuffield Department of Medicine, NIHR Biomedical Research Centres, University of Oxford, Oxford, UK; 5 Pandemic Sciences Institute, Nuffield Department of Medicine, University of Oxford, Oxford, UK; 6 Division of Infectious Diseases, David Geffen School of Medicine, University of California, Los Angeles, Los Angeles, CA USA; 7 Kirby Institute, University of New South Wales, Sydney, New South Wales Australia; 8 Immunology Disease Area, Novartis BioMedical Research, Basel, Switzerland; 9 Kennedy Institute of Rheumatology, University of Oxford, Oxford, UK; 10 University Children’s Hospital, John Radcliffe Hospital, Oxford, UK; 11 Janssen Research & Development, Immunology Translational Sciences and Medicine, La Jolla, CA USA; 12 Department of Pathology, University of Cambridge, Cambridge, UK; 13 Wellcome Sanger Institute, University of Cambridge, Cambridge, UK; 14 NIHR Oxford Biomedical Research Centre, University of Oxford, Oxford, UK; 15 Peter Medawar Building for Pathogen Research, Nuffield Department of Medicine, University of Oxford, Oxford, UK

**Keywords:** Translational immunology, Coeliac disease

## Abstract

The immune–epithelial–stromal interactions underpinning intestinal damage in celiac disease (CD) are incompletely understood. To address this, we performed single-cell transcriptomics (RNA sequencing; 86,442 immune, parenchymal and epithelial cells; 35 participants) and spatial transcriptomics (20 participants) on CD intestinal biopsy samples. Here we show that in CD, epithelial populations shifted toward a progenitor state, with interferon-driven transcriptional responses, and perturbation of secretory and enteroendocrine populations. Mucosal T cells showed numeric and functional changes in regulatory and follicular helper-like CD4^+^ T cells, intraepithelial lymphocytes, CD8^+^ and γδ T cell subsets, with skewed T cell antigen receptor repertoires. Mucosal changes remained detectable despite treatment, representing a persistent immune–epithelial ‘scar’. Spatial transcriptomics defined transcriptional niches beyond those captured in conventional histological scores, including CD-specific lymphoid aggregates containing T cell–B cell interactions. Receptor–ligand spatial analyses integrated with disease susceptibility gene expression defined networks of altered chemokine and morphogen signaling, and provide potential therapeutic targets for CD prevention and treatment.

## Abstract (web summary)

FitzPatrick et al. use single-cell RNA and spatial transcriptomics to dissect the immune microenvironments within the small bowel mucosa in celiac disease.

## Main

Celiac disease (CD) is a common gastrointestinal disorder affecting 1–2% of European and North American populations, in which small intestinal inflammation and damage are driven by aberrant adaptive immune responses to gluten^1^. The only treatment is a lifelong gluten-free diet (GFD). There is an unmet therapeutic need for those living with CD, including refractory CD, where ongoing tissue damage occurs despite a GFD^2^.

A strong genetic component drives CD, dominated by HLA-DQ2 and HLA-DQ8 (ref. ^3^), with association studies identifying over 40 non-HLA genomic loci, implicating over 100 candidate genes and a role for immunoregulatory mechanisms^4^. Murine models implicate viral infection as a trigger of loss of tolerance driving CD pathogenesis^5,6^, a hypothesis supported by epidemiological studies^7^.

CD pathophysiology is multifactorial with several cell types implicated^8–10^. Dietary gluten is deamidated by tissue transglutaminase 2, and deamidated gluten peptides presented via HLA-DQ2/HLA-DQ8 to CD4^+^ T cells^11^. Gluten-specific CD4^+^ T cells possess a distinct type 1 helper T (T_H_1)/follicular helper T (T_FH_) cell phenotype, emphasizing the importance of T cell–B cell interactions^12^. Tissue plasma and B cells may present gluten peptides via HLA-DQ^13,14^. Subsequent stimulation of disease-specific plasma cells drives anti-tissue transglutaminase and anti-deamidated gliadin peptide antibody production.

Gluten-specific T cells are necessary but not sufficient to generate mucosal damage^15^. The mechanisms by which this response leads to tissue architectural change are incompletely understood. Intraepithelial lymphocytes (IELs), mainly CD8^+^ T IELs, are highly enriched in CD, likely driven by epithelial and myeloid-derived interleukin (IL)-15, in combination with CD4^+^ T cell-derived IL-2, IL-21 and interferon gamma (IFNγ)^15,16^. IELs may be directly involved in EC killing in a T cell antigen receptor (TCR)-independent manner, via NKG2C and NKG2D and their epithelial ligands MICA and HLA-E^17,18^. However, the transcriptional state and involvement of TCR signaling in these CD8^+^ T cell populations remains unclear.

While novel treatments are under development^2^, recent therapeutic trials targeting gluten degradation, gluten-specific CD4^+^ T cell tolerance and IL-15 have been unsuccessful^19–22^. However, therapies including tissue transglutaminase inhibitors and inducers of immune tolerance have shown promise^23–25^.

Single-cell transcriptomics have redefined cellular landscapes in the gastrointestinal tract^26,27^, offering insights into CD immunopathology^28^. Recent studies have sought to understand the cellular basis of CD using mass cytometry, including studies of refractory CD^29^, gluten-specific T cells^12^, and mucosal and circulating T cells^30^. Single-cell RNA sequencing (scRNA-seq) has been used to study mucosal immune cells^31^, T cells^32^, circulating immune cells^33^ and mucosal plasma cells^34^.

Here, we combined single-cell and spatial transcriptomics to define the network of intestinal immune, epithelial and parenchymal cell populations in adults and children with CD. Our description of spatially localized immune–parenchymal interactions driving inflammation and remodeling of the mucosa, and with specific disease-associated T cell subsets occupying distinct mucosal niches, will facilitate identification of therapeutic targets.

## Results

**Fig. 1:**
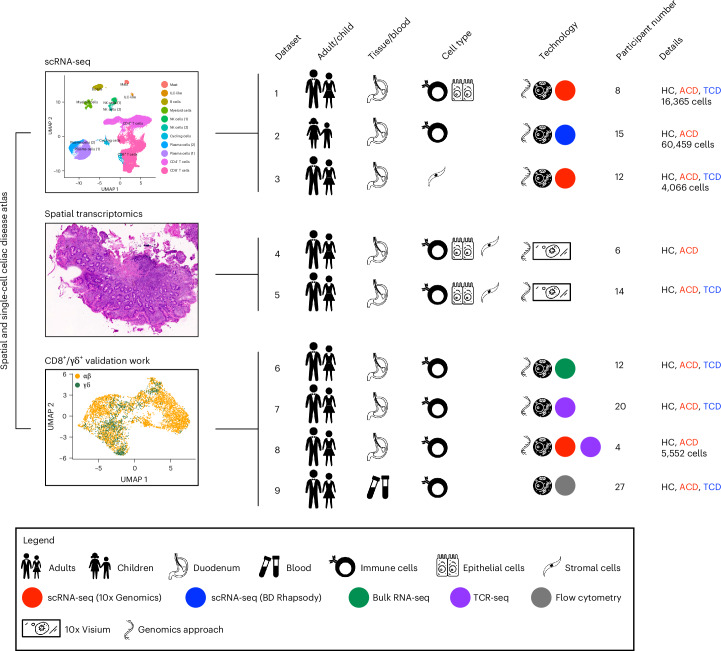
Study schematic. Schematic of scRNA-seq, RNA-seq, TCR-seq, spatial transcriptomics, and flow cytometry experiments and datasets. Dataset 1: ECs and total mucosal CD45^+^ cells were isolated from intestinal biopsy samples before scRNA-seq library preparation using the 10x Genomics platform. Dataset 2: total mucosal CD45^+^ cells were isolated from intestinal biopsy samples before combined targeted scRNA-seq and multiplex surface antibody characterization using the BD Rhapsody platform. Dataset 3: scRNA-seq (10x Genomics) was performed on intestinal stromal and endothelial cells. Datasets 4 and 5: OCT-embedded frozen duodenal biopsy samples were sectioned and used for spatial transcriptomics (10x Visium). Datasets 6 and 7: mucosal CD8^+^ T cells were isolated before bulk RNA-seq and TCR-seq. Dataset 8: mucosal CD8^+^ and γδ^+^ T cells were isolated before scRNA-seq library preparation using the 10x Genomics platform. Dataset 9: flow cytometry of circulating CD8^+^ T cells. Study participant numbers and disease characteristics, as well as cell numbers after the quality-control pipeline, are indicated. ILC, innate lymphoid cell; HC, healthy controls; ACD, active celiac disease; TCD, treated celiac disease.

We generated scRNA-seq profiles of duodenal epithelial, immune and parenchymal populations from 35 participants: 21 with CD (16 children, 5 adults) and 14 controls (5 children, 9 adults; Fig. 1 and Supplementary Table 1). We used complementary single-cell techniques for adult and pediatric datasets, with 86,442 cells sequenced. In adults (datasets 1 and 3), we performed scRNA-seq (10x Genomics) on epithelial, immune (Supplementary Fig. 1a,b), stromal and endothelial cells. In children (dataset 2), we performed targeted scRNA-seq (BD Rhapsody; 504 targeted gene primer pairs) and surface protein expression (79 oligonucleotide-conjugated antibodies) on intestinal immune cells (Supplementary Fig. 1c,d and Supplementary Tables 2 and 3).

### The duodenal epithelial compartment in CD

**Fig. 2:**
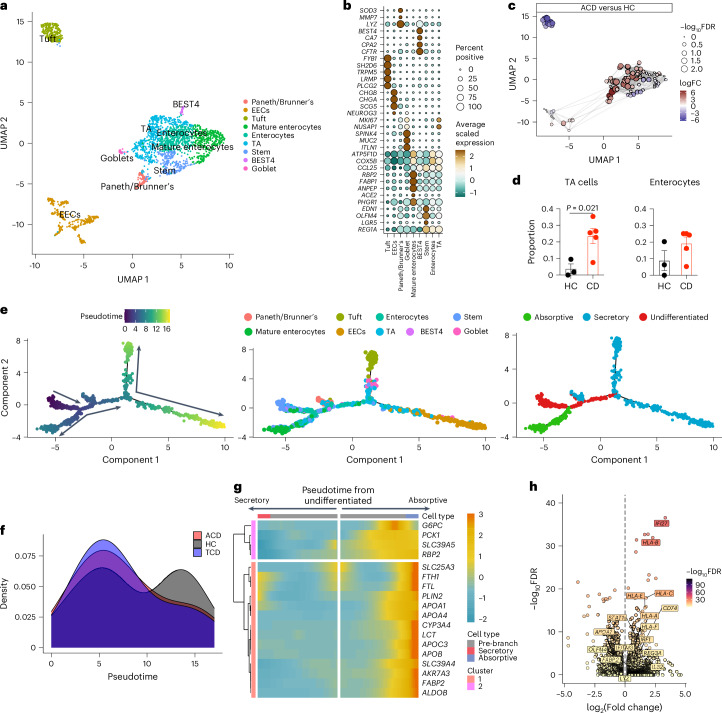
ECs in CD. **a**, UMAP plot of small intestinal epithelial EPCAM^+^ cells in HCs (*n* = 3) and in participants with CD (*n* = 5). **b**, Bubble plot showing the expression of selected genes defining specific cluster identities. Scaled gene expression indicated by color; proportion of cells expressing the gene indicated by bubble size. **c**, Local neighborhood enrichment of *EPCAM*^+^ cells in ACD versus HCs. Color indicates enrichment (log fold change (FC)) of cells in ACD versus HCs in that UMAP neighborhood; size of dot indicates false discovery rate (FDR)-adjusted −log_10_ values. **d**, TA cells (left) and early enterocytes (right) in HCs and CD, as a proportion of total *EPCAM*^+^ cells. **e**, Pseudotime trajectory of gene expression of *EPCAM*^+^ ECs, colored by pseudotime axis (left), cluster identity (middle) and lineage (right). Arrows indicate putative direction of cell differentiation. **f**, Density of cells along pseudotime trajectory axis split by disease state: ACD (red), TCD (blue) and HCs (gray). **g**, Smoothed heat map showing expression of selected genes related to intestinal absorption along pseudotime trajectories relating to secretory (toward left) and absorptive (toward right) lineage. **h**, Volcano plot displaying differentially expressed gene transcripts between HCs and ACD in total ECs. **d**, Unpaired two-tailed *t*-test. Data are presented as mean values ± s.e.m.

**Extended Data Fig. 1:**
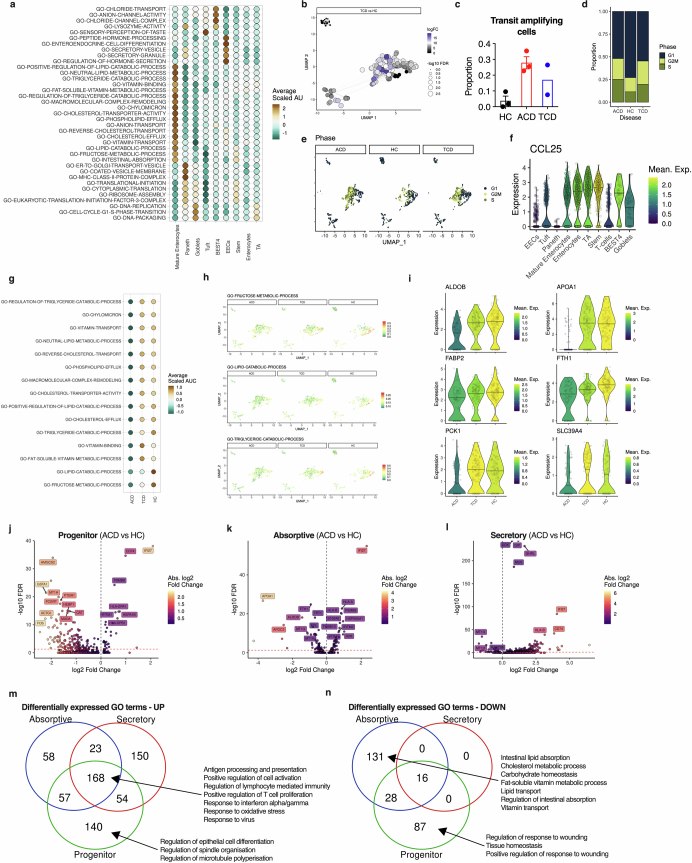
Epithelial cells in celiac disease. (**a**) Bubble plot showing the expression of selected GO term gene sets defining specific cluster identities and functions. Gene set expression indicated by colour. (**b**) Local neighbourhood enrichment of *EPCAM*^+^ cells in treated CD vs healthy controls (HC). Colour indicates enrichment (log fold change) of cells in active CD vs HC in that UMAP neighbourhood, size of dot indicates –log_10_FDR. (**c**) Transit amplifying cells in HC (n = 3), active CD (n = 3), and treated CD n = 2), as a proportion of total *EPCAM*^+^ cells (mean ± SEM). (**d**) Proportion of *EPCAM*^+^ cells in predicted cell cycle states (G1, G2M, S), stratified by disease state. (**e**) UMAP overlay of predicted cell cycle states (G1, G2M, S), stratified by disease state. (**f**) Violin plot of CCL25 expression by epithelial cell type. (**g**) Expression of GO term gene sets associated with metabolic and absorptive function in mature enterocytes, stratified by disease state. (**h**) UMAP overlay of expression of selected GO term gene sets associated with metabolic and absorptive function, stratified by disease state. (**i**) Violin plots of expression of genes associated with absorptive function in mature enterocytes, stratified by disease state. (**j–l**) Volcano plots displaying differentially expressed gene transcripts between HC and active CD in the three cell lineages: epithelial progenitors (**i**), absorptive cells (**j**), and secretory cells (**k**). (**m, n**) Venn diagrams showing shared differentially expressed GO term gene sets between progenitor, absorptive, and secretory lineages, either upregulated (**l**) or downregulated (**m**) in active CD compared to HC.

We analyzed *EPCAM*^+^ epithelial populations from dataset 1. Nine transcriptionally distinct epithelial cell (EC) clusters were identified, representing progenitor, secretory and absorptive lineages along the developmental progression of the crypt–villus axis (Fig. 2a,b, Extended Data Fig. 1a and Supplementary Table 4). BEST4 enterocytes (*BEST4*^+^*CA7*^+^*CPA2*^+^), first identified in the colon^35^, were seen, expressing *CFTR* and showing chloride channel activity (Fig. 2b and Extended Data Fig. 1a). Goblet cells (*ITLN1*^+^*MUC2*^+^*SPINK4*^+^) and tuft cells (*PLCG2*^+^*TRPM5*^+^*IRAG2*^+^) were also identified.

A *LYZ*^*+*^ Paneth cell-like population (*MMP7*^+^*REG1A*^+^*SOD3*^+^*PLA2G2A*^+^) was also identified (Fig. 2a,b), although defensin gene expression was not detected. This population expressed *PGC*, mucins including *MUC5AC*, *MUC1* and *MUC6* and *AQP5*, suggesting it also contained Brunner’s gland cells or ectopic gastric pyloric gland cells. This cell type was enriched in active celiac disease (ACD; Fig. 2c,d), perhaps in response to IFNγ. Thus, this population could represent inflammation-driven gastric cell metaplasia^36^.

Transit-amplifying (TA) cells were increased in CD, along with enrichment of uniform manifold approximation and projection (UMAP) areas corresponding to EC progenitors (stem cells, TA cells and early enterocytes; Fig. 2c,d). This persisted in treated celiac disease (TCD; Extended Data Fig. 1b,c). In parallel, more actively cycling ECs were observed in ACD and TCD (Extended Data Fig. 1d,e).

Pseudotime analyses identified epithelial developmental trajectories, from undifferentiated progenitor states toward absorptive and secretory lineages (Fig. 2e). In CD, ECs were shifted to earlier pseudotime states, with loss of mature ECs (Fig. 2f). *CCL25*, encoding the ligand for CCR9 (implicated in CD pathogenesis^37^), was expressed predominantly by progenitor cells (Fig. 2b and Extended Data Fig. 1f).

We examined putative EC functions through functional gene-set analysis (Extended Data Fig. 1a), identifying functions of secretory Paneth-like/Brunner’s gland cells (secreted protein and vesicle pathways), BEST4 enterocytes (chloride/anion channel activity), tuft cells (taste perception) and enteroendocrine cells (EECs; peptide hormone processing/secretion). Mature enterocytes expressed key metabolic and macronutrient catabolic pathways, and active transport and absorption mechanisms. Early ECs and TA cells did not express these pathways. Absorptive function genes were limited to cell states at the end of absorptive epithelium pseudotime trajectories, consistent with EC development along the crypt–villus axis (Fig. 2g). Notably, gene sets related to lipid, carbohydrate, cholesterol, vitamin and iron processing and absorption were all downregulated in mature enterocytes in ACD (Extended Data Fig. 1g–i). These transcriptional changes normalized in TCD, although some pathways, including fructose metabolism and lipid catabolism, remained reduced (Extended Data Fig. 1h). Overall, absorptive capacity is reduced in ACD not simply by reduction in villus surface area, but through a relative increase of EC progenitors lacking absorptive machinery, and pathway downregulation in mature enterocytes.

ECs in ACD upregulated multiple antigen-presentation molecules, including classical HLA class I and class II genes (except *HLA-DQ*) and nonclassical genes including *HLA-E* and *HLA-F* (Fig. 2h). Interferon-stimulated genes (types I and II) dominated the epithelial response, including *STAT1* (Fig. 2h and Supplementary Table 5). The major disease-associated responses were observed in all EC lineages (Extended Data Fig. 1j–l), including antigen-presentation pathways, type I/II interferon responses, lymphocyte-mediated immunity and cytotoxicity and cell adhesion regulation (Extended Data Fig. 1m,n).

Some transcriptional changes were cell-type specific. *IL32* was highly expressed in ACD by mature enterocytes (Extended Data Fig. 1k), perhaps regulated by interferons. The reduction of fatty acid catabolism/transport (*APOA1*, *FABP2*), metal ion transport (iron: *FTH1*, *FTL*; zinc: *SLC39A4*) and carbohydrate metabolism (*ALDOB*, *PCK1*) was restricted to absorptive lineages, mainly mature enterocytes (Extended Data Fig. 1k,n). Progenitor cells upregulated genes associated with cell division and differentiation, and downregulated those associated with tissue repair and homeostasis (Extended Data Fig. 1m,n). Secretory lineages showed increased expression of gut hormone genes, *LYZ*, and chemokines (*CXCL17*, *CXCL2*; Extended Data Fig. 1l).

**Extended Data Fig. 2:**
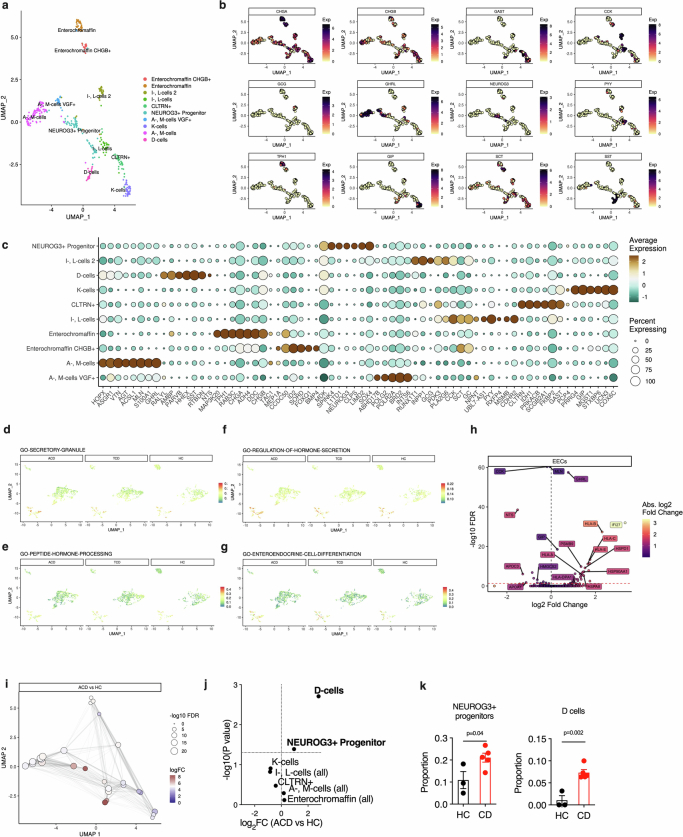
Enteroendocrine cells in celiac disease. (**a**) UMAP plot of intestinal enteroendocrine cells (EECs) in healthy controls (HC) and celiac disease (CD) (n = 8). (**b**) UMAP plots overlaid with expression of genes identifying enteroendocrine cell types. (**c**) Bubble plot showing the expression of selected genes defining specific cluster identities. Gene expression indicated by colour, proportion of cells expressing the gene indicated by bubble size. (**d–g**) UMAP overlay of expression of selected GO term gene sets associated with peptide hormone secretion and enteroendocrine differentiation, stratified by disease state. (**h**) Volcano plot displaying differentially expressed gene transcripts between HC and active CD in enteroendocrine cells. (**i**) Local neighbourhood enrichment of enteroendocrine cells in treated CD vs HC. Colour indicates enrichment (log fold change) of cells in active CD vs HC in that UMAP neighbourhood, size of dot indicates –log_10_FDR. (**j**) Volcano plot of enteroendocrine subset proportion (log2 fold change) between CD and HC. (**k**) NEUROG3+ progenitors and D cells in HC (n = 3) and CD (n = 5), as a proportion of total enteroendocrine cells (mean ± SEM). Unpaired two-tailed t-test.

The duodenum, where CD inflammation predominates, has sensory and neurohormonal functions. We extended EEC clustering, revealing multiple transcriptional states, including NEUROG3^+^ progenitors and EEC subtypes, which showed similar CD-related transcriptional changes to other ECs (Extended Data Fig. 2). EEC proportions altered in CD, with increases in NEUROG3^+^ progenitor cells and somatostatin-producing D cells (Extended Data Fig. 2i–k).

### Intestinal T_FH_-like CD4^+^ T cells are increased in CD

**Fig. 3:**
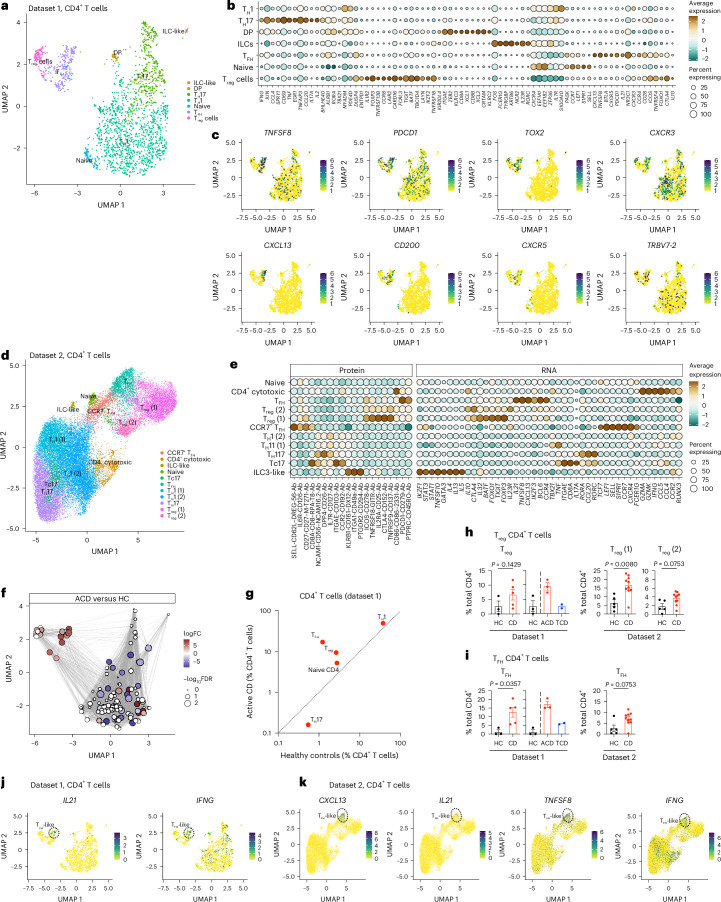
CD4^+^ T cells in CD. **a**–**c**, Intestinal CD4^+^ T cells in health and CD in dataset 1 (adult—10x Genomics). **a**, UMAP plot of intestinal CD4^+^ T cells in health and CD (*n* = 8). **b**, Bubble plot showing the expression of selected genes defining specific cluster identities. Scaled gene expression indicated by color; proportion of cells expressing the gene indicated by bubble size. **c**, CD4^+^ T cell UMAP plots overlaid with expression of *TNFSF8*, *PDCD1*, *TOX2*, *CXCR3*, *CXCL13*, *CD200*, *CXCR5* and *TRBV7-2*. Intestinal CD4^+^ T cells in health and CD in dataset 2 (pediatric—BD Rhapsody; **d**–**f**). **d**, UMAP plot of intestinal CD4^+^ T cells in health and CD (*n* = 15). **e**, Bubble plot showing the expression of selected genes and proteins defining specific cluster identities. Scaled gene/protein expression indicated by color; proportion of cells expressing the gene/protein indicated by bubble size. **f**, Local neighborhood enrichment of CD4^+^ cells in ACD versus HCs (dataset 1). Color indicates enrichment (log fold change) of cells in ACD versus HCs in that UMAP neighborhood; size of dot indicates −log_10_FDR. **g**, Scatterplot of mean proportion (± s.e.) of CD4^+^ T cell clusters in HCs (*n* = 3) versus ACD (*n* = 5) in dataset 1. Clusters above the line of unity are enriched in ACD. **h**,**i**, T_reg_ (**h**) and T_FH_ (**i**) CD4^+^ T cell populations in HCs and CD, as a proportion of total CD4^+^ T cells in dataset 1 (HCs *n* = 3, ACD *n* = 5) and dataset 2 (HCs *n* = 5, ACD *n* = 10). **j**, UMAP plot of CD4^+^ T cells in dataset 2, overlaid with *IL21* and *IFNG* expression. **k**, UMAP plot of CD4^+^ T cells in dataset 1, overlaid with *CXCL13*, *IL21*, *IFNG* and *TNFSF8* expression. **h**,**i**, Two-sided Mann–Whitney test. Data are presented as mean values ± s.e.m. Ab, antibody; Tc17, IL17^+^CD8^+^ T cells; DP, CD4^+^CD8^+^ double positive cells.

**Extended Data Fig. 3:**
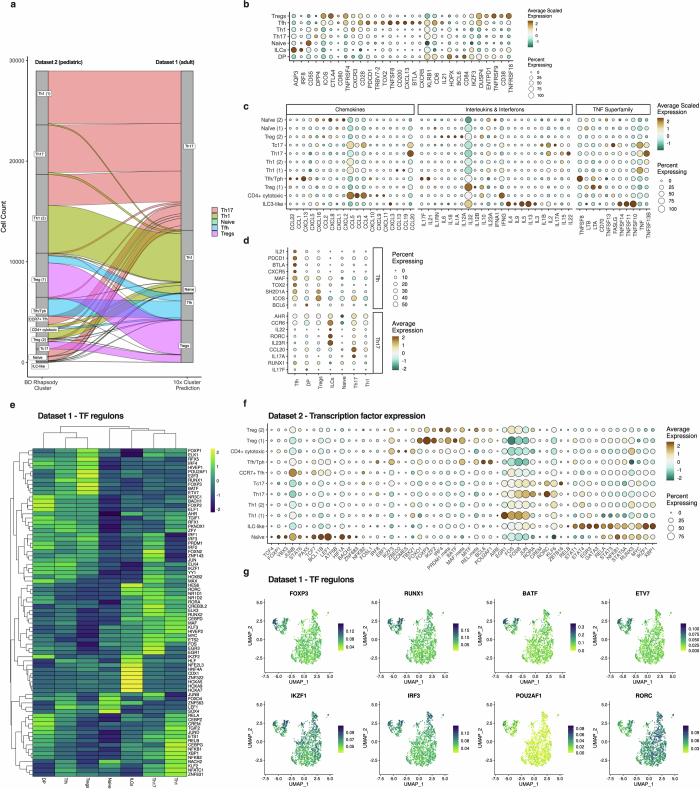
CD4^+^ T cells. (**a**) Sankey plot showing predicted analogous CD4^+^ T-cell clusters between Dataset 1 and Dataset 2 datasets. (**b**) Bubble plot showing the expression of selected genes associated with CD4^+^ T-cells clusters, including genes previously associated with gluten-specific CD4^+^ T-cells (Christophersen et al, 2019). Gene expression indicated by colour, proportion of cells expressing the gene indicated by bubble size. (**c**) Bubble plot of expression of chemokine, cytokine, and TNF family member genes by CD4^+^ T-cell clusters in Dataset 2 dataset. Gene expression indicated by colour, proportion of cells expressing the gene indicated by bubble size. (**d**) Bubble plot showing expression of canonical Tfh and Th17 gene signatures in CD4 + T cell clusters. (**e**) Heatmap of transcription factor (TF) regulon expression by CD4^+^ T-cell clusters in Dataset 1. (**f**) Transcription factor gene expression by CD4^+^ T-cell clusters in Dataset 2. (**g**) UMAP plots of CD4^+^ T-cells in Dataset 1, overlaid with expression of selected TF regulons.

In adults (dataset 1), CD4^+^ T cells formed subsets dominated by T_H_1-polarized and IL-17-producing helper T (T_H_17)-polarized effectors, as well as small naive and FOXP3^+^ regulatory populations (Fig. 3a–c and Supplementary Table 6). There was a cluster of T_FH_-like CD4^+^ T cells expressing *PDCD1*, *BTLA*, *CD28*, *ICOS* and intermediate *CXCR5*. Dataset 2 (pediatric) contained analogous subsets (Extended Data Fig. 3a), including CD31^+^CR2^+^ recent thymic emigrants^38^, a CCR7^+^ T_FH_-like subset and the T_FH_-like subset expressing PD1, ICOS, CTLA4, BTLA and CD161 at the protein level (Fig. 3d,e).

This T_FH_-like population in adults and children showed similar phenotypic profiles to those of gut-resident gluten-specific CD4^+^ T cells in CD^12^ (Extended Data Fig. 3b), and expressed *TOX2*, *CD200*, *IL21* and *CXCL13*. The cluster showed enrichment of *TRBV7-2*, a V-gene enriched in gluten-specific CD4^+^ T cell HLA-DQ2.5^+^ TCR repertoires^39^. T_reg_ and T_FH_-like CD4^+^ T cells were increased in ACD in adults and children (Fig. 3f–i).

T cell populations showed distinct cytokine and chemokine expression patterns (Extended Data Fig. 3c). The CD-associated T_FH_-like population, showed high *CXCL13* and *IL21* expression, with *IFNG* and *IL21* coexpression (Fig. 3j,k), similarly to gluten-specific T cells^12,40^. T_FH_-like cells expressed *TNFSF8*, *CCL1*, *CCL22* and *CXCL10*, as well as *IL17F* (Extended Data Fig. 3c). *IL17F* expression was not seen in the *IL17A*^+^*RORC*^+^*IL23R*^+^*CCR6*^+^ T_H_17 population, nor did the T_H_17 cluster show *TRBV7-2* enrichment (Extended Data Fig. 3b,d).

Oral gluten challenge in CD drives rapid circulating cytokine responses, including IL-2, CXCL8, CXCL10 and IL-6 (ref. ^41^). *CXCL8* expression was highest in CCR7^+^ T_FH_ CD4^+^ T cells, *CXCL10* was detected in T_FH_-like CD4^+^ T cells, while *IL6* was detected in T_reg_ cells (Extended Data Fig. 3c). *IL2* expression was low within the CD4^+^ compartment, as expected without gluten challenge.

We examined transcription factor (TF), and regulon expression within CD4^+^ subsets, with canonical TFs and regulons of T_H_17 and T_reg_ cell function expressed as expected (Extended Data Fig. 3e–g). *IKZF1* and its regulon were upregulated in T_FH_-like cells, with intermediate expression of *RUNX1*, *BATF* and *IRF3*.

### Myeloid and B cell lineages in CD

**Extended Data Fig. 4:**
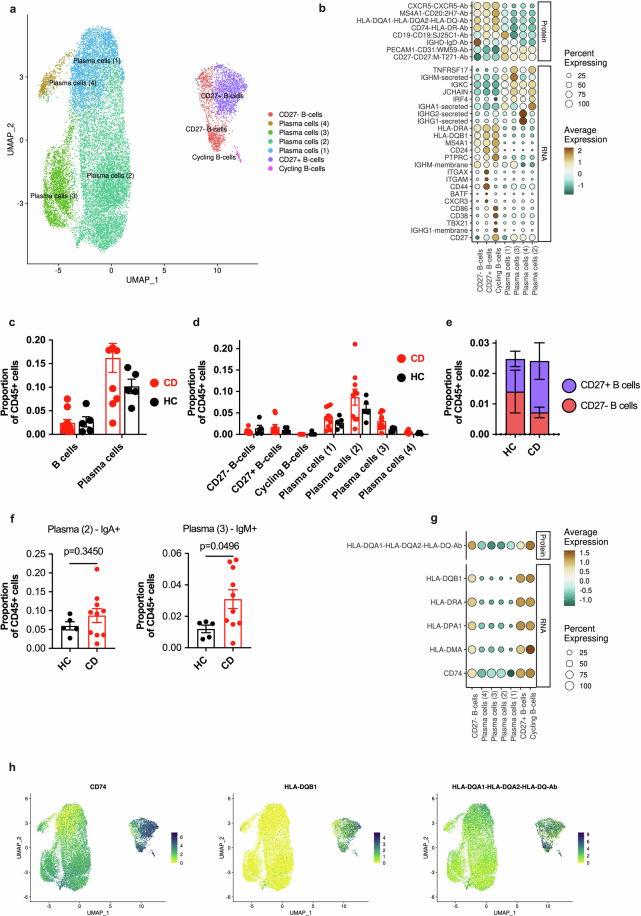
B cell and plasma cells in celiac disease. B cell and plasma cell clusters were examined in the Dataset 2 (pediatric) dataset (BD Rhapsody) in healthy controls (HC) (n = 5) and CD (n = 10). (**a**) UMAP plot of intestinal B and plasma cell clusters. (**b**) Bubble plot showing the expression of selected genes and surface proteins defining specific cluster identities. Gene/protein expression indicated by colour, proportion of cells expressing the gene indicated by bubble size. (**c**) Intestinal B cell and plasma cell frequencies in HC and CD, as a proportion of total CD45^+^ cells. (**d**) Intestinal B cell and plasma cell cluster frequencies in HC and CD, as a proportion of total CD45^+^ cells. (**e**) Proportion of intestinal CD27- and CD27 + B cells in HC and CD, as a proportion of total CD45^+^ cells. (**f**) Proportion of intestinal IgA and IgM plasma cells in HC and CD, as a proportion of total CD45^+^ cells. (**g**) Bubble plot showing the expression of genes and proteins related to MHC class II expression. (**h**) UMAP plots overlaid with the expression of genes CD74 and HLA-DQB1, and the surface protein expression of HLA-DQA1/A2. (**c**-**f**) Data presented as mean ± SEM. (**f**) Unpaired two-tailed t-test.

We examined B cell lineages in dataset 2 (pediatric; Extended Data Fig. 4a,b). Both IgA^+^ and IgM^+^ plasma cells were increased in CD^42,43^ (Extended Data Fig. 4c–f). A population of CXCR5^+^ B cells (*MS4A1*^+^*CD19*^+^*CD20*^+^) were present, with a shift toward the CD27^+^ memory B cell phenotype in CD.

Gene signatures of age-related B cells (an inflammation-associated population in autoimmune disease^44^), including *ITGAM*, *ITGAX*, *CD86* and *BATF*, were expressed most highly in CD27^+^ B cell populations, while a key age-related B cell TF, *TBX21*, was highly expressed in cycling B cells (Extended Data Fig. 4b). HLA class II gene and protein expression, specifically *HLA-DQ*, was highest in CD27^+^ and cycling B cells (Extended Data Fig. 4g,h).

Intestinal myeloid cell populations are impacted by CD and may be involved in antigen presentation and oral tolerance^5^. Myeloid cells (dataset 2) formed 11 transcriptionally distinct clusters, including macrophages, conventional dendritic cells and plasmacytoid dendritic cells (Supplementary Fig. 2a–c). HLA-DQ expression was highest on macrophage populations, particularly CD163^+^ cells. In contrast to prior studies^45^, CD163^+^ macrophages were reduced in ACD, with expansion of a conventional dendritic cell 2 population, which showed increased IL-1B expression (Supplementary Fig. 2d,e).

### Tissue-resident memory CD8^+^ T cells in CD

**Fig. 4:**
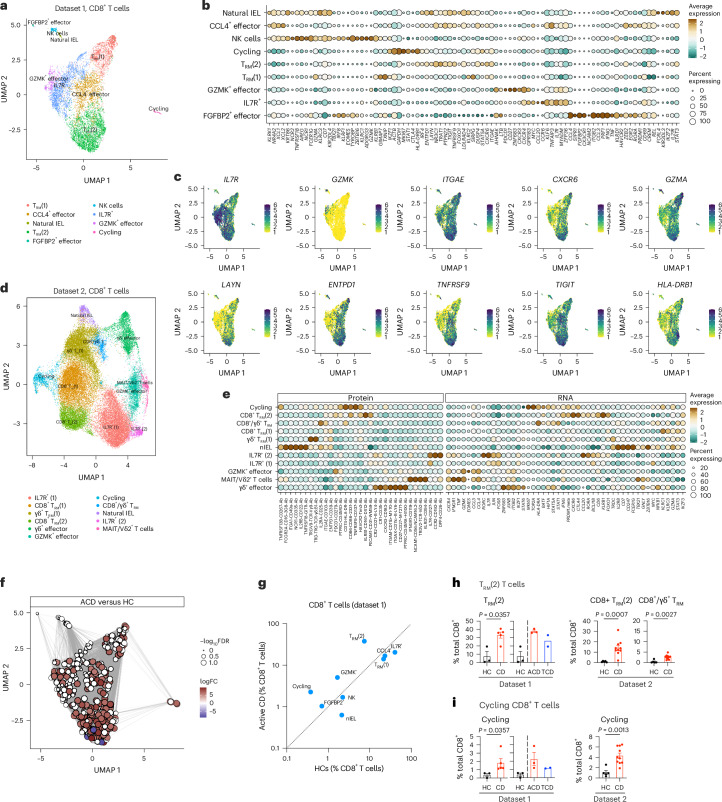
CD8^+^ T cells in CD. **a**–**c**, Dataset 1 intestinal CD8^+^ T cells in health and CD (adult—10x Genomics). **a**, UMAP plot of intestinal CD8^+^ T cells in health and CD (*n* = 8). **b**, Bubble plot showing the expression of selected genes defining specific cluster identities. Scaled gene expression indicated by color; proportion of cells expressing the gene indicated by bubble size. **c**, UMAP plots overlaid with expression of *IL7R*, *GZMK*, *ITGAE*, *CXCR6*, *GZMA*, *LAYN*, *ENTPD1*, *TNFRSF9*, *TIGIT* and *HLA-DRB1*. Dataset 2 intestinal CD8^+^ T cells in health and CD (pediatric—BD Rhapsody; **d**–**f**). **d**, UMAP plot of intestinal CD8^+^ T cells in health and CD (*n* = 15). **e**, Bubble plot showing the expression of selected genes and proteins defining specific cluster identities. Gene/protein expression indicated by color; proportion of cells expressing the gene/protein indicated by bubble size. **f**, Local neighborhood enrichment of CD8^+^ cells in ACD versus HCs (dataset 1). Color indicates enrichment (log fold change) of cells in ACD versus HCs in that UMAP neighborhood; size of dot indicates −log_10_FDR. **g**, Scatterplot of mean proportion (± s.e.) of CD8^+^ T cell clusters in HCs (*n* = 3) versus ACD (*n* = 5). Clusters above the line of unity are enriched in ACD. **h**,**i**, T_RM_(2) (**h**) and cycling (**i**) CD8^+^ T cell phenotype populations in HCs and CD, as a proportion of total CD8^+^ T cells in dataset 1 (HCs *n* = 3, ACD *n* = 5) and dataset 2 (HCs *n* = 5, ACD *n* = 10). **h**,**i**, Two-sided Mann–Whitney test. Data are presented as the mean values ± s.e.m. nIEL, natural intraepithelial lymphocyte.

**Extended Data Fig. 5:**
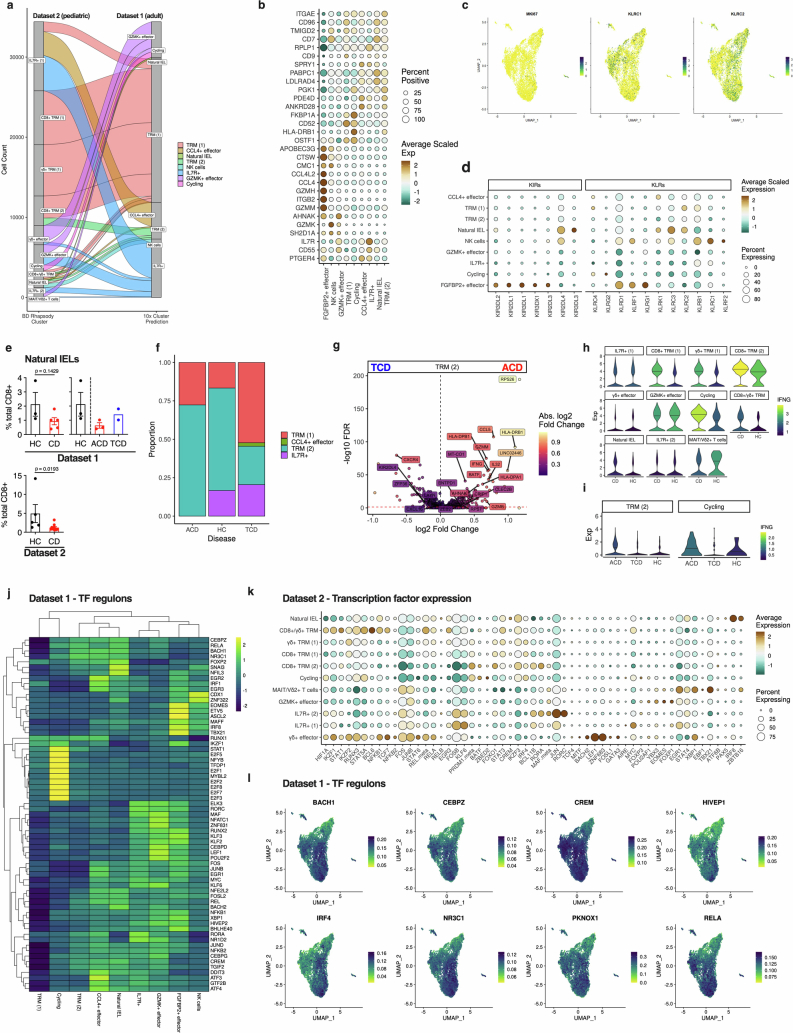
CD8^+^ T cells in celiac disease. (**a**) Sankey plot showing predicted analogous CD8^+^ T-cell clusters between Dataset 1 and Dataset 2 datasets. (**b**) Bubble plot showing the expression of selected genes associated with CD8^+^ T_RM_ cell subsets^55^. Gene expression indicated by colour, proportion of cells expressing the gene indicated by bubble size. (**c**) UMAP plot of CD8^+^ T-cells in Dataset 1, overlaid with *MKI67, KLRC1*, and *KLRC2* expression. (**d**) Bubble plot showing the expression of KIRs and NKRs associated with previously described CD8^+^ T-cell subsets in CD. (**e**) Natural IEL populations in HC (n = 3) and CD (n = 5), as a proportion of total CD8^+^ T-cells in Dataset 1 (above) and Dataset 2 (below) (mean ± SEM). (**f**) Stacked plot of predicted CD8^+^ T-cell cluster origin of cycling CD8^+^ T-cells, stratified by disease state. (**g**) Volcano plot displaying differentially expressed gene transcripts between active and treated CD in TRM(2) CD8^+^ cells. (**h**) Violin plot of *IFNG* expression in CD8+ clusters in Dataset 2, split by disease state. (**i**) Violin plot of *IFNG* expression in T_RM_(2) and cycling CD8+ clusters in Dataset 1, split by disease state. (**j**) Heatmap of transcription factor (TF) regulon expression by CD8^+^ T-cell clusters in Dataset 1. (**k**) Transcription factor gene expression by CD8^+^ T-cell clusters in Dataset 2. (**l**) UMAP plots of CD8^+^ T-cells in Dataset 1, overlaid with expression of selected TF regulons. (**e**) Unpaired two-tailed t-test.

Intestinal CD8^+^ T cells showed considerable heterogeneity in transcriptional states, with multiple tissue-resident memory CD8^+^ T (T_RM_) cells, including an *ITGAE*^−^*IL7R*^+^ population, a *CCL4*^+^*CD69*^+^*ITGAE*^+^ population and two subsets of *ITGAE*^+^ T_RM_ cells (Fig. 4, Extended Data Fig. 5a and Supplementary Table 7). These aligned with gene signatures defining subsets of bona fide human T_RM_ cells^46^. *FGFBP2*^+^ effectors aligned with previously described *ITGB2*^+^*ITGAE*^−^ T_RM_ cells, while T_RM_(1), T_RM_(2) and cycling subsets aligned with CD103^+^ T_RM_ cells (Extended Data Fig. 5b). *CCL4*^+^ and *IL7R*^+^ populations likely represent intermediate states in T_RM_ cell development. Small natural IEL and cycling *MKI67*^+^ populations were seen (Extended Data Fig. 5b,c). Analogous CD8^+^ T cell subsets were seen in dataset 2 (Fig. 4d,e and Extended Data Fig. 5a), with additional resolution for tissue-resident γδ T cells, and innate-like T cells (mucosal-associated invariant T cells and Vδ2Vγ9^+^ cells).

We analyzed subsets relevant to CD, including natural killer (NK)-receptor expressing IELs^17,18^ and killer-cell immunoglobulin-like receptor (KIR)-positive CD8^+^ T cells^47^. *KLRC1* (NKG2A) was expressed by CCL4^+^ cells, while *KLRC2* (NKG2C) was expressed by resident IL7R^+^, T_RM_(1) and T_RM_(2) subsets (Extended Data Fig. 5c,d). Inhibitory KIR molecule expression was confined to a small FGFBP2^+^ effector population.

T_RM_(2) and cycling populations were enriched in ACD, but not T_RM_(1) cells (Fig. 4f,g). T_RM_(2) cells were rare in health, but increased to form 20–40% of CD8^+^ T cells in ACD, which persisted in TCD (Fig. 4h). Natural IELs were reduced in ACD (Extended Data Fig. 5e). Cycling CD8^+^ T cells increased to form 2–4% of cells in ACD (Fig. 4i). Most cycling cells showed a T_RM_(2) phenotype (Extended Data Fig. 5f).

**Fig. 5:**
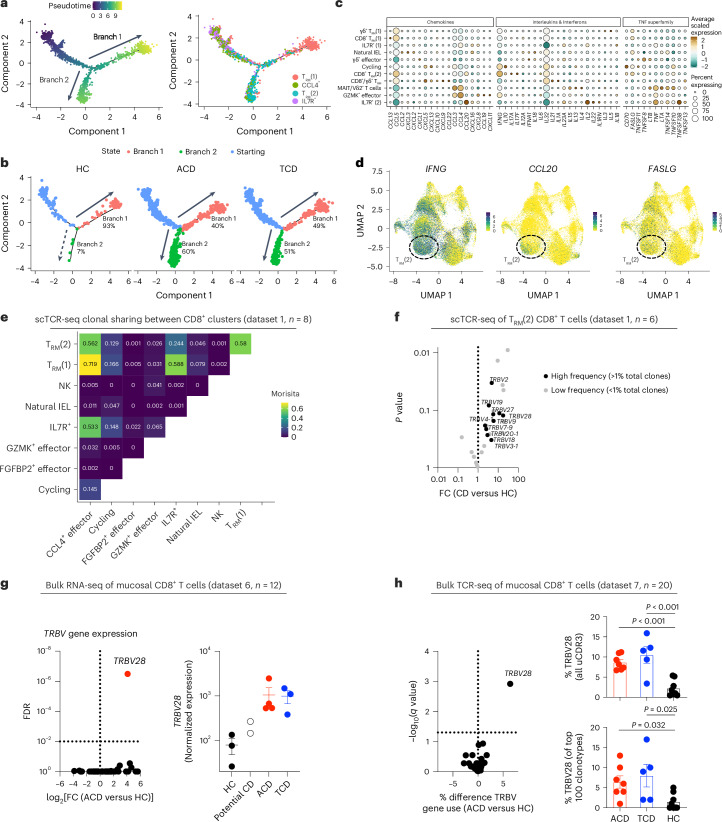
CD8^+^ T cells in CD. **a**, Pseudotime trajectory of gene expression of tissue-resident CD8^+^ T cell clusters (dataset 1—adult), colored by pseudotime axis (left) and cell cluster (right). Arrows indicate direction of differentiation. **b**, Pseudotime trajectory, split by disease state, and colored by differentiation branch. The proportion of CD8^+^ T_RM_ cells differentiating down branches 1 and 2 in each disease state is indicated. **c**, Bubble plot of expression of chemokine, cytokine and TNF family member genes by CD8^+^ T cell clusters in dataset 2 (pediatric). Scaled gene expression indicated by color; proportion of cells expressing the gene indicated by bubble size. **d**, UMAP plots of CD8^+^ T cells in dataset 2 (pediatric), overlaid with *IFNG*, *CCL20* and *FASLG* expression. **e**, TCR clonal overlap (Morisita–Horn) between CD8^+^ T cell clusters in dataset 1. **f**, Volcano plot of TRBV segment usage within the TCR repertoire of T_RM_(2) cells between HCs and CD. Black, high-frequency TRBV segments used by >1% of total clones; gray, low-frequency TRBV segments used by <1% of total clones. **g**, Volcano plot of TRBV segment gene expression (left) and normalized expression of TRBV28 (right) in bulk RNA-seq data from sorted intraepithelial CD8^+^ T cells (dataset 3; HCs *n* = 3, ACD *n* = 4, TCD *n* = 3, potential CD *n* = 2). **h**, Volcano plot of TRBV segment usage (left), and proportion of unique CDR3β clonotypes (right above) and proportion of top 100 most common clonotypes (right, below) using the TRBV28 V segment in bulk TCR-seq of CD8^+^ mucosal T cells in HCs and CD (dataset 4; HCs *n* = 8, ACD *n* = 7, TCD *n* = 5). **f**, Negative binomial model without multiple comparisons. **g**, Negative binomial model with Benjamini–Hochberg multiple testing. **h**, One-way analysis of variance with Holm–Sidak’s multiple-comparisons test.

**Extended Data Fig. 6:**
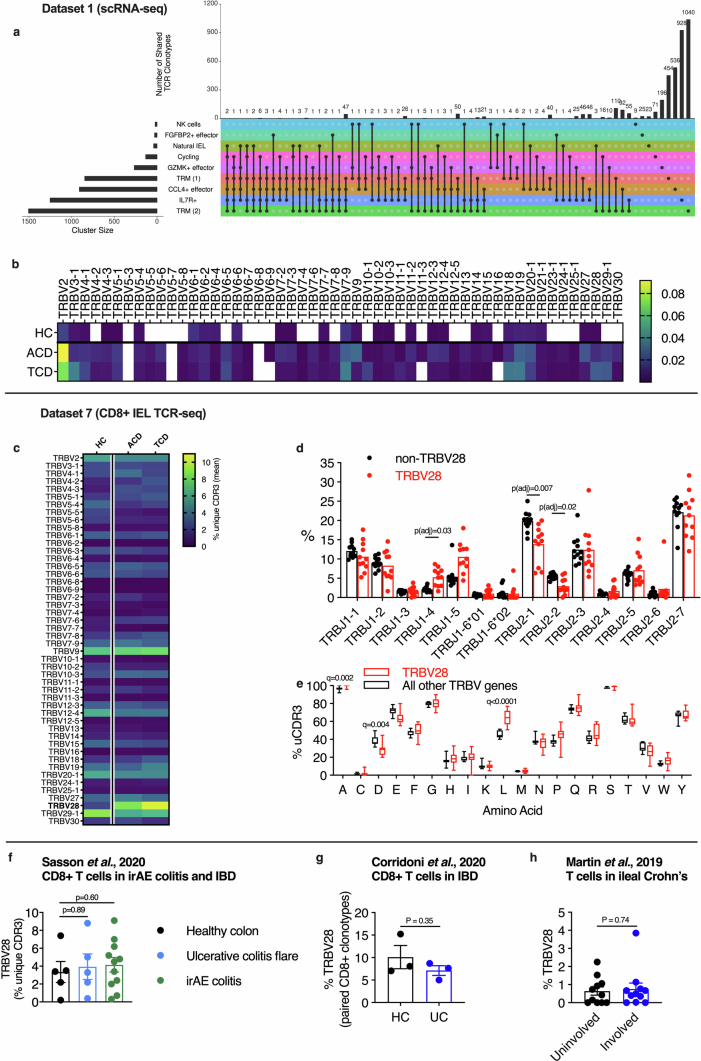
CD8^+^ T cells and TCR repertoires in celiac disease. (**a, b**) Single-cell TCR-seq of CD8^+^ T-cells in Dataset 1. (**a**) Upset plot of T-cell clonotype overlap between CD8^+^ T-cell clusters. (**b**) TRBV segment usage in TCR clonotypes in T_RM_(2) CD8^+^ T-cells. (**c–e**) TCR-seq of sorted mucosal CD8^+^ T-cells (n = 20; Dataset 4). (**c**) TRBV gene usage by unique CDR3 clonotypes in intestinal CD8^+^ T-cell TCR repertoires in health and celiac disease. (**d**) TRBJ gene usage by TRBV28 (red) and non-TRBV28 (black) clonotypes in intestinal CD8^+^ T-cell TCR repertoires in CD (n = 12) (mean ± SEM). (**e**) Amino acid usage within CDR3 sequences of TRBV28 (red) and non-TRBV28 (black) clonotypes in intestinal CD8^+^ T-cell TCR repertoires in CD (n = 12) (median, IQR, range). (**f**) Proportion of unique CDR3β clonotypes using the TRBV28 V-gene in health (n = 5), active ulcerative colitis (n = 5), and irAE colitis (n = 11) (mean ± SEM). Data from Sasson and colleagues^60^. (**g**) Proportion of unique paired CD8^+^ clonotypes using the TRBV28 V-gene in health (n = 3) and active ulcerative colitis (n = 3) (mean ± SEM). Data from Corridoni and colleagues^61^. (**h**) Proportion of unique paired T cell clonotypes using the TRBV28 V-gene in uninflamed (n = 11) and inflamed (n = 11) biopsies in ileal Crohn’s disease (mean ± SEM). Data from Martin and colleagues^62^. (**c, d, f**) Two-way ANOVA with Holm-Sidak’s multiple comparisons test. (**e**) Multiple two-tailed t-tests with Benjamini, Krieger, and Yetutieli two-stage step up control of multiple comparisons with FDR < 0.01. (**g**,**h**) Mann-Whitney two-tailed test.

As T_RM_(2) CD8^+^ T cells were increased in proportion and proliferating in ACD, we profiled them in depth (Fig. 5a–e and Extended Data Fig. 6). T_RM_(2) CD8^+^ T cells showed a CD103^+^ tissue-resident phenotype, high *GZMA* and absent *GZMK* expression, along with high expression of CXCR6, activation markers (*HLA-DR*) and genes expressing co-stimulatory and co-inhibitory molecules (*TIGIT*, *TNFRSF9* (4-1BB), *ENTPD1* (CD39) and *LAYN* (Fig. 4b,c). Comparison of T_RM_(2) cells in ACD versus TCD showed increased expression of activation markers and increased effector function with *IFNG*, *GZMB* and *IL32* expression (Extended Data Fig. 5g).

We examined pseudotime trajectories of tissue-resident clusters with TCR repertoire clonal sharing (T_RM_(1), T_RM_(2), IL7R^+^ and CCL4^+^ effectors) to infer putative differentiation pathways (Fig. 5a,b,e). The pseudotime trajectory showed two branches, formed predominantly of T_RM_(1) cells in branch 1 and T_RM_(2) cells in branch 2, developing from IL7R^+^ and CCL4^+^ populations (Fig. 5a). While branch 1 cells were seen in both controls and CD, strikingly, branch 2 was almost totally restricted to ACD and TCD (Fig. 5b).

We examined cytokine, chemokine and TF expression by CD8^+^ T cell subsets. The predominant CD8^+^ sources of *IFNG* were CD8^+^ T_RM_(2) and cycling clusters (Fig. 5c,d and Extended Data Fig. 5g–i). These populations also expressed the chemokine *CCL5*, *CD70* and *FASLG*. Natural IELs (reduced in ACD), produced *CCL2*, *CXCL2*, *CXCL3*, *IL12*, *IL18* and type I interferon. T_RM_(1) and T_RM_(2) CD8^+^ subsets showed distinct TF and regulon profiles; T_RM_(2) cells were associated with the TF regulons BACH1, CEBPZ, CREM, IRF4 and NR3C1 and TF expression of *RORA*, *PRDM* and *FOXO1* (Extended Data Fig. 5j–l).

### CD8^+^ TCR repertoires are altered in CD

CD8^+^ T cell-induced epithelial damage is thought to be mediated via TCR-independent mechanisms. We hypothesized that CD8^+^ T cell TCR repertoires would be similar in health and disease. Single-cell TCR sequences were examined, which showed expected clonal overlap between tissue-resident populations (Fig. 5e and Extended Data Fig. 6a). Cluster TRBV gene usage was examined between health and CD. Several high-frequency TRBV segments (>1% total clones) were overrepresented in CD (Fig. 5f and Extended Data Fig. 6b). However, statistical power was limited due to low clonotype numbers.

Consequently, we sorted intraepithelial CD8^+^ T cells from 12 adults with and without CD (dataset 3) and performed bulk RNA-seq. This showed significant enrichment of one TRBV segment, *TRBV28*, enriched in ACD and TCD, but not controls (Fig. 5g). *TRBV28* was the high-frequency V segment with the highest fold change for enrichment in CD within the T_RM_(2) population (Fig. 5f).

We validated this by performing bulk TCR repertoire sequencing on 1,068,814 mucosal CD8^+^ T cells from 20 donors with and without CD (dataset 4). Again, *TRBV28* was highly upregulated in CD, forming 10% of unique CDR3 sequences in ACD and TCD, versus 2% in controls (Fig. 5h). *TRBV28* was also enriched within the top 100 most expanded clonotypes. No association with TRAV usage was seen. Clonotypes containing *TRBV28* in CD paired with multiple TRBJ segments, and showed altered CDR3 amino acid usage, with enrichment of germline-encoded and non-germline-encoded leucine residues (Extended Data Fig. 6c–e).

We examined bulk TCR repertoires of intestinal CD8^+^ T cells of colonic and small intestinal biopsy samples from three separate studies examining non-CD inflammatory gastrointestinal conditions^48–50^. There was no signal for enrichment of *TRBV28* gene usage in these disease settings (Extended Data Fig. 6f–h).

**Extended Data Fig. 7:**
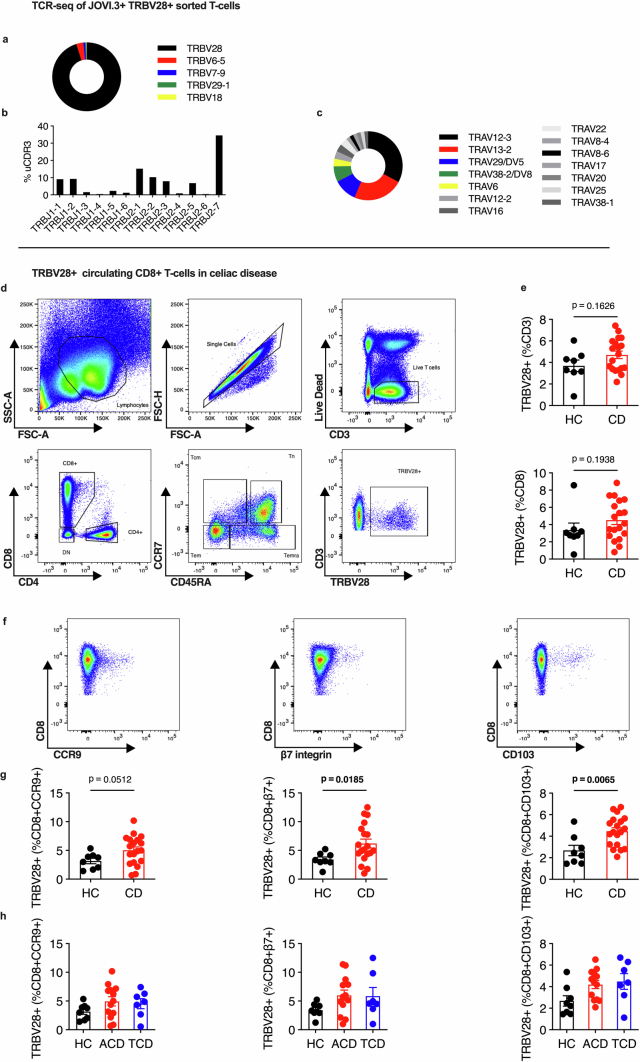
TRBV28 expression by circulating CD8^+^ T cells in celiac disease. (**a**) TRBV gene usage (proportion of unique CDR3 clonotypes) of sorted CD3^+^ JOVI.3^+^ T-cells, determined by bulk TCR-seq. (**b**) TRBJ gene usage of TRBV28 clonotypes from sorted CD3^+^ JOVI.3^+^ T-cells. (**c**) TRAV gene usage (proportion of unique CDR3 clonotypes) of sorted CD3^+^ JOVI.3^+^ T-cells. Flow cytometry examining TRBV28 surface protein expression on circulating CD8 + T-cells in healthy controls (n = 8), active CD (n = 13), and treated CD (n = 7). (**d**) Representative flow cytometry gating strategy. (**e**) TRBV28 expression by total CD3^+^ T-cells (left) and total CD8^+^ T-cells (right) in peripheral blood in health and CD. (**f**) Representative plots of staining for CCR9, β7-integrin, and CD103. (**g, h**) TRBV28 expression by CCR9^+^ (left), β7-integrin^+^ (middle), and CD103^+^ (right) CD8^+^ T-cells in peripheral blood, categorised by health and CD (**g**), and by health, active and treated CD (**h**). (**e**,**g**) Mann-Whitney two-tailed test. (**e**,**g**,**h**) Mean ± SEM shown.

We hypothesized that differences in mucosal CD8^+^ TCR repertoire/phenotype may be mirrored within gut-homing CD8^+^ T cells in the circulation, as seen following gluten challenge^30^. We examined TRBV28 usage by circulating CD8^+^ T cells using flow cytometry (dataset 9). Using TCR sequencing (TCR-seq), we validated the specificity of the TRBV28-specific antibody clone (JOVI.3; Extended Data Fig. 7a–c). As expected, there was no difference in the fraction of TRBV28^+^ cells in total peripheral CD3^+^ or CD8^+^ T cell compartments in participants with and without CD. However, within CD8^+^ T cell populations expressing gut-specific chemokines (CCR9) or integrins (CD103/β-integrin), the fraction of TRBV28^+^ cells was increased in ACD and TCD (Extended Data Fig. 7d–h).

### γδ T cell populations and TCR repertoires are altered in CD

**Extended Data Fig. 8:**
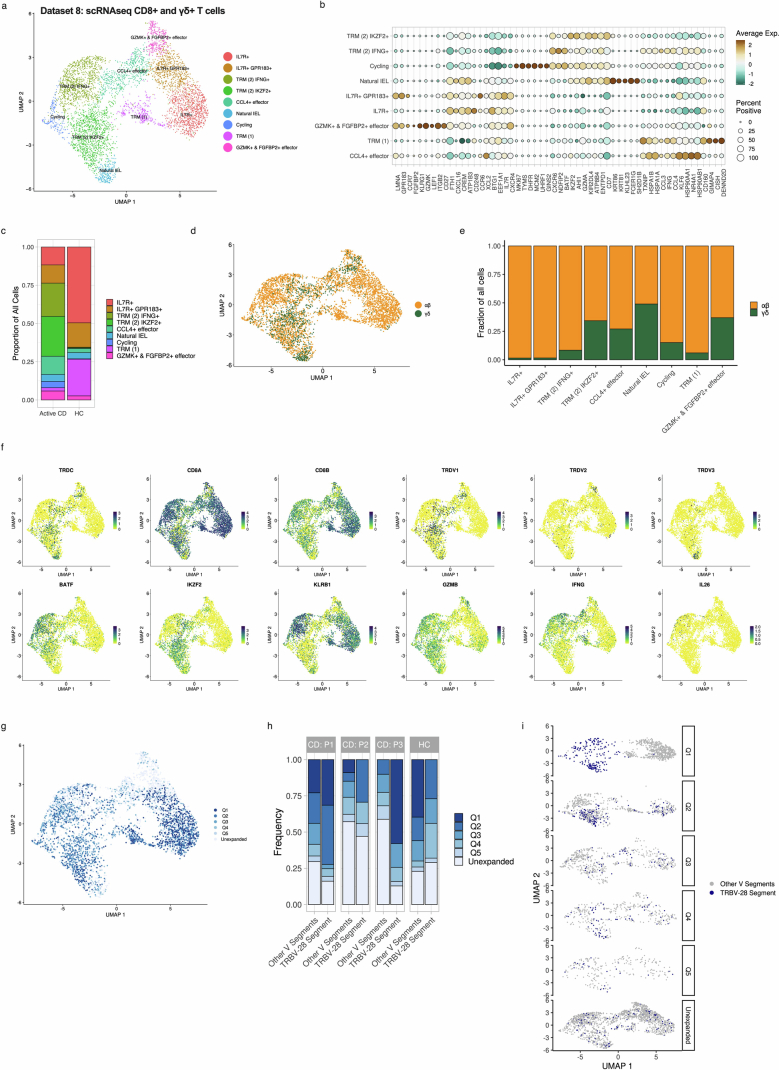
Single-cell analysis of mucosal αβ CD8^+^ and γδ T cells in celiac disease. Sorted intestinal γδ+ and αβ + CD8^+^ T-cells in CD. (**a**) UMAP plot of intestinal CD4^+^ T-cells in health and CD (n = 4). (**b**) Bubble plot showing the expression of selected genes defining specific cluster identities. Scaled gene expression indicated by colour, proportion of cells expressing the gene indicated by bubble size. (**c**) Proportion of transcriptional clusters in HC and CD. (**d**) UMAP overlay and (**e**) stacked plots showing the proportion of γδ + T cells in each transcriptional cluster. (**f**) UMAP plot overlaid with the expression of genes of interest. (**g**) UMAP plot overlaid with the clonal expansion of TCR clones. Clonal expansion expressed as quintiles, with Q1 showing the most expanded clones. (**h**) Frequency of quintiles of clonal expansion in TCR sequences expressing TRBV28. (**i**) UMAP plot overlaid with TRBV28-expressing CD8 + TCR clonotypes, split by quintiles of clonal expansion.

Intraepithelial duodenal γδ T cells are increased in CD, although their role is unclear^32,51^. We analyzed a further dataset of 5,552 sorted intestinal CD8^+^ αβ^+^ and γδ^+^ T cells (dataset 8; Extended Data Fig. 8). Clustering of cell transcriptional states recapitulated the key populations described above (Extended Data Fig. 8a,b). As previously, the T_RM_(2) population (in this case split into *IFNG*^+^ and *IKZF2*^+^ subpopulations) was increased in ACD, along with cycling T cells (Extended Data Fig. 8c).

γδ T cells showed overlapping transcriptional profiles with mucosal CD8^+^ αβ T cells, albeit with enrichment within specific clusters (Extended Data Fig. 8d,e). γδ T cells were most enriched within a natural IEL phenotype cluster and the GZMK^+^/FGFBP2^+^ effector populations, and were also present in the CCL4^+^ effector and IKZF2^+^ T_RM_(2) population. γδ T cells were uncommon within IFNG^+^ T_RM_(2) and cycling clusters. *TRDV1* and *TRDV3* expression was higher in the CCL4^+^, IKZF2^+^ T_RM_(2) and natural IEL populations, with *TRDV3* in particular enriched in the natural IEL cluster (Extended Data Fig. 8f).

We analyzed the TCR repertoire of CD8^+^ T cells in this dataset. The T_RM_ and IL7R^+^ clusters showed greatest clonal expansion (Extended Data Fig. 8g). In all participants with CD, TRBV28-containing clonotypes were more clonally expanded than their non-TRBV28 counterparts. TRBV28^+^ clonotypes were enriched in the top quintile of expanded clones, which were almost exclusively found within the T_RM_(2) and cycling clusters (Extended Data Fig. 8h,i).

We validated these findings through bulk RNA-seq of sorted intestinal αβ^+^ CD8^+^ and γδ T cells from participants with and without CD (dataset 6). Gene-set enrichment analysis of CD8^+^ T cell gene expression in ACD showed upregulation of TCR activation gene sets, and enrichment of cluster marker gene sets from T_RM_(2) and cycling populations (Supplementary Fig. 3a–c), with upregulation of *CXCR6*, *ENTPD1* and *MKI67* (Supplementary Fig. 3d). CD8^+^ T cells showed upregulation of *IFNG* and *IL26* (Supplementary Fig. 3e). There was a shift from *KLRC1* (NKG2A) to *KLRC2* (NKG2C) expression, but *KLRK1* (NKG2D) expression was not increased. Inhibitory KIRs were upregulated in this dataset, consistent with recent findings^47^. Gene expression between health and CD was different in γδ^+^ and αβ^+^ CD8^+^ T cells (Supplementary Fig. 3f,g). *IFNG* and *MKI67* expression were not increased to the same extent in γδ^+^ T cells, nor were T_RM_(2) IFNG^+^ cluster markers like *ENTPD1*. There were also differences in NK cell receptor changes, KIRs, *PDCD1* and *TYROBP*, a natural IEL marker (Supplementary Fig. 3h).

Bulk γδ TCR repertoire sequencing (dataset 7) revealed a skewed TRGV repertoire, with reduced TRGV4 and increased TRGV3 use in ACD (Supplementary Fig. 4a,b), which persisted after treatment, as previously described^51^. Most TRD CDR3 sequences were private; however, increased sequence sharing was noted between ACD repertoires (Supplementary Fig. 4c), with longer shared CDR3 sequences in ACD (Supplementary Fig. 4d). Previously reported CD-associated TRDV CDR3 motifs^30^, were increased in ACD; however, we were unable to replicate the previously described association between the TRDV H-J1 motif and CD^51^ (Supplementary Fig. 4e–i).

### Immunoepithelial changes persist in TCD

Samples from adults with TCD (GFD with good symptomatic, serological and histological response) were included in scRNA-seq, bulk RNA-seq and TCR-seq experiments (Supplementary Table 1). We hypothesized that cell-type and transcriptional changes would normalize with treatment. However, many biological changes persisted.

Specifically, EC changes including increased TA cell proportions and cycling cells (Extended Data Fig. 1b–e) and the shift toward progenitor states (Fig. 2f) persisted despite treatment. EEC changes also persisted. However, absorptive function gene expression within mature enterocytes had predominantly normalized, aside from the ongoing reduction in fructose metabolism and lipid catabolism (Extended Data Fig. 1g).

While T_FH_-like CD4^+^ T cells and T_reg_ cells returned toward control levels on treatment, the CD8^+^ compartment remained perturbed, with reduced natural IELs and increases in T_RM_(2) CD8^+^ T cells. However, the T_RM_(2) population showed reduced activation in TCD (lower *IFNG*, *IL32* and pro-inflammatory markers). Intestinal CD8^+^ TCR repertoire changes remained (specifically *TRBV28* enrichment), as did increased circulating TRBV28^+^ gut-homing CD8^+^ T cells.

### Stromal and endothelial populations in CD

We next analyzed duodenal stromal and endothelial populations in CD (dataset 3; Supplementary Fig. 5a–d). Annotation of stromal populations based on previous descriptions showed S1, S2 and S3 fibroblasts, as well as myofibroblasts, with S1 fibroblasts most common in the duodenum. The pro-inflammatory S4 phenotype seen in colonic inflammatory bowel disease^52^ was not seen. Differential gene expression and gene-set enrichment analysis showed upregulation of interferon-induced genes including *STAT1*, the major histocompatibility complex class II invariant chain (*CD74*), and *SLIT2*, encoding a secreted protein involved in intestinal homeostasis (Supplementary Fig. 5e,f). Analysis of endothelial cells revealed arterial, capillary, venous and lymphatic populations, with upregulation of interferon-stimulated genes in CD (Supplementary Fig. 5g–i).

### Spatial transcriptomics reveals LAs in CD

**Fig. 6:**
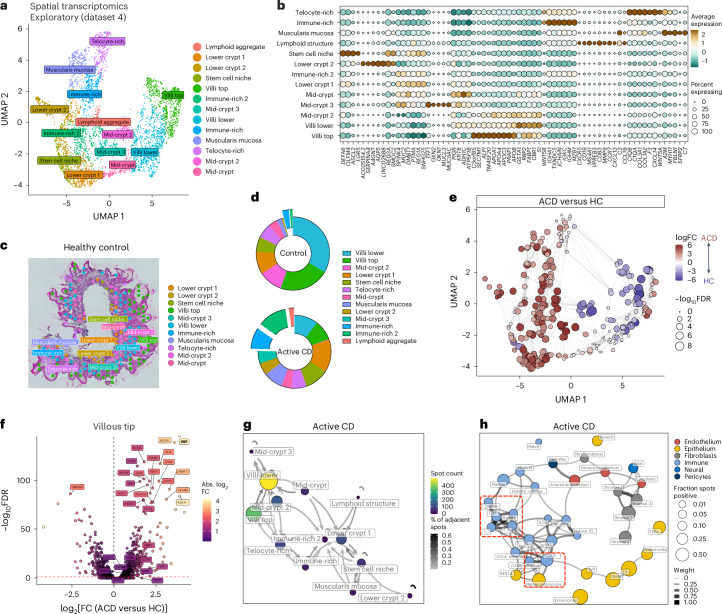
Spatial transcriptomics of the intestinal mucosa reveals localized patterns of immune cell distribution. **a**, UMAP overlay of all spatial transcriptomics tissue-covered spots with transcriptome-driven clustering analysis, colored by region. **b**, Bubble plot showing the expression of selected genes defining spatial regions. Scaled gene expression indicated by color; proportion of cells expressing the gene indicated by bubble size. **c**, Visualization of transcriptionally distinct spatial regions overlaid on representative HC tissue section. **d**, Proportion of intestinal mucosa formed in different regions in HCs (above) and ACD (below). Immune-rich and LA regions are highlighted. **e**, Local neighborhood enrichment of intestinal mucosal regions in ACD versus HCs. Color indicates enrichment (log fold change) of cells in ACD versus HCs in that UMAP neighborhood. **f**, Volcano plot of differential gene expression between HCs and ACD within villus tip spatial regions. **g**, The spatial relationships between different regions in ACD can be visualized using a network plot. Regions that are more likely to be adjacent to another region are connected by arrows colored by the percentage of adjacent spots. Region size is indicated by size and color of the region circle. **h**, Integrating scRNA-seq reference data localizes single-cell transcriptomes to spatial regions. These data are used to generate network plots visualizing colocalization of cell types together in ACD. Cell-type nodes close together and linked by connecting lines are more often located in the same spots. In ACD, mature enterocytes colocalize with T_RM_(2) CD8^+^ T cells (lower red box), while T_FH_-like CD4^+^ T cells localize with B cells, T_reg_ cells and plasma cells (upper red box).

We next performed spatial transcriptomics on duodenal biopsy samples (dataset 4; Fig. 6). Spatial transcriptomics showed 13 transcriptionally distinct regions within the mucosa, representing compartments of the crypt–villus axis (stem cell niche, lower-crypt and mid-crypt regions and villus zones), stromal cell-rich regions, several lamina propria regions with immune cell infiltrates dominated by plasma cell signatures and lymphoid aggregates (LAs; Fig. 6a,b). In health, the epithelial villus compartments dominated; these were reduced in ACD (Fig. 6c–e). These villus regions expressed absorptive function genes, predominantly in the most mature villus compartment (Fig. 6b and Supplementary Fig. 6b). In contrast, immune-rich regions and LAs were greatly expanded in ACD (Fig. 6d,e). These regions were themselves spatially organized, with LAs closely associated with lower-crypt and immune-rich regions, and telocyte-rich regions with villus structures (Fig. 6g).

These immune-rich regions and LAs showed gene expression patterns associated with B cells (*CD19*, *MS4A1* (CD20)), plasma cells (*IGHM*, *IGHA1*, *TXNDC5*) and T cells (*CD3D*, *CCR7*, *CXCL13*). Signals of cellular proliferation (*MKI67*, *REG1A*) were highly localized in specific clusters. In health, cellular proliferation was limited to stem-cell and lower-crypt regions, while in ACD, proliferative markers were more dispersed, including in immune-rich and LA regions (Supplementary Fig. 6c,d).

In ACD, villus top regions showed increases in interferon-stimulated genes, markers of proliferation, and *IL32* (Fig. 6f), analogous to epithelial scRNA-seq results. TCR genes, tissue-residency markers (*ITGAE*, *CXCR6*, *KLRB1*), and cytotoxic CD8^+^ markers (*GZMA*, *KLRD1*, *KLRK1*) were increased in ACD, suggesting tissue-resident cytotoxic CD8^+^ T cell enrichment in villi.

We integrated spatial transcriptomics with scRNA-seq data to predict cell-type locations in mucosa (Supplementary Figs. 6e–j and 7). In ACD, TA cell signatures expanded from crypt bases to most villus regions, while mature EC signatures were restricted to superficial epithelial layers. In ACD, LAs showed highly localized enrichment of T_FH_-like CD4^+^ T cell (*CXCR5*, *CXCL13*) and B cell (*CD19*, *MS4A1*; Fig. 6b and Supplementary Fig. 7a) signatures. Plasma cell signatures were expanded in neighboring immune-rich regions. In ACD, CD8^+^ T_RM_(2) cell signatures were highly enriched in villus tip regions, colocalized with mature enterocytes (Fig. 6h and Supplementary Figs. 6h–j and 7b).

**Fig. 7:**
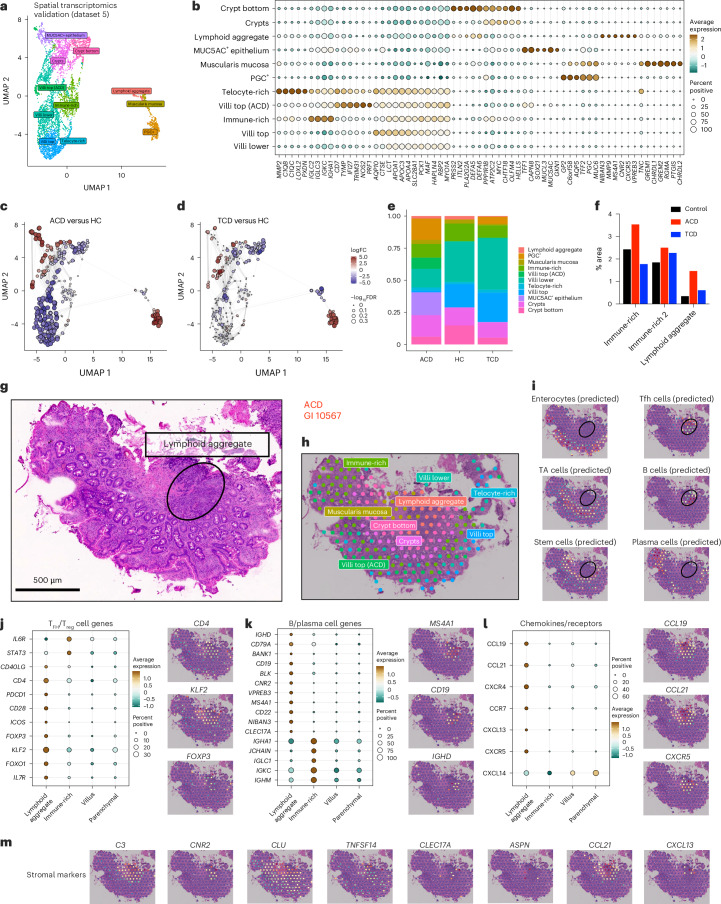
T_FH_–B cell interactions are highly localized in LAs in the celiac lesion. **a**, UMAP overlay of all spatial transcriptomics tissue-covered spots with transcriptome-driven clustering analysis, colored by region. **b**, Bubble plot showing the expression of selected genes defining spatial regions. Scaled gene expression indicated by color; proportion of cells expressing the gene indicated by bubble size. **c**,**d**, Local neighborhood enrichment of intestinal mucosal regions in ACD versus HCs (**c**) and TCD versus HCs (**d**). Color indicates enrichment (log fold change) of cells in CD versus HCs in that UMAP neighborhood. **e**, Proportion of intestinal mucosa formed in different regions in HCs, ACD and TCD. **f**, Proportion of immune-rich and LA regions in HCs, ACD and TCD. **g**,**h**, Detailed examination of a representative LA in ACD (seen in 5/10 CD sections). **g**, Hematoxylin and eosin (H&E)-stained section of duodenal biopsy with LA circled. **h**, Spatial regions overlaid onto the section show the LA near the lower-crypt/stem-cell niche region, and near the muscularis mucosa. **i**, Predicted cell-type locations in regions overlaid onto the section. **j**–**l**, Bubble plots of gene expression within LAs and other regions, paired with gene expression overlaid onto an ACD section with LA, including T_FH_/T_reg_ cell gene signatures (**j**), B/plasma cell gene signatures (**k**), and chemokines and associated receptors (**l**). **m**, Stromal cell gene expression overlaid onto a representative ACD section with LA.

**Extended Data Fig. 9:**
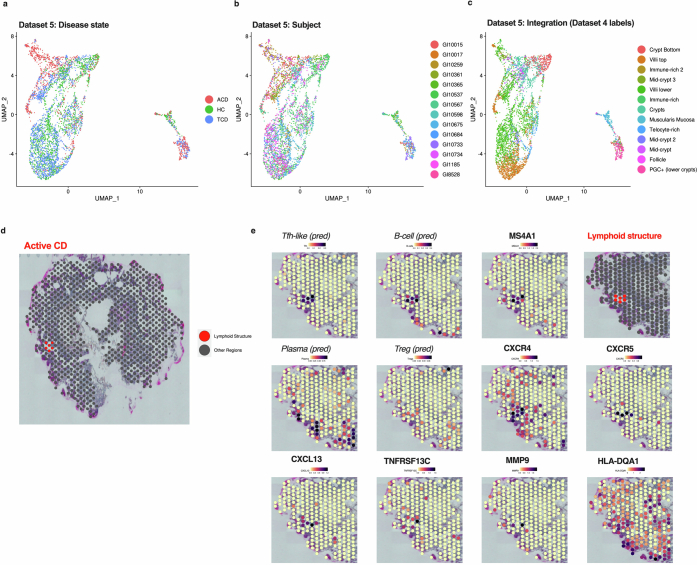
Spatial transcriptomics. (**a–c**) Dataset 5 spatial transcriptomics descriptive data and integration with Dataset 4. (**a**) UMAP overlaid with disease state. (**b**) UMAP overlaid with study subject ID. (**c**) UMAP overlaid with Dataset 4 region labels. (**d, e**) Cell type predictions and spatially restricted gene expression within lymphoid aggregates within the duodenal mucosa in CD. Lymphoid aggregate regions are highly localised in intestinal biopsies from CD subjects (**b**). Cell type predictions (**c**) indicate Tfh-like CD4^+^ T-cells, as well as B cells, are present in these regions, with plasma cells and Tregs surrounding these regions. This is supported by the expression of *MS4A1* (CD20), *CXCR4, CXCR5* and *CXCL13*.

To further study LAs in CD, we performed further spatial transcriptomics experiments on duodenal biopsy samples in participants with and without CD (dataset 5; Fig. 7 and Extended Data Fig. 9a–c). Analysis of spatial regions recapitulated our description of key transcriptional regions in the duodenal mucosa (Fig. 7a,b and Extended Data Fig. 9c), with enrichment of proliferating areas at crypt bases, MUC5AC^+^ and PGC^+^ epithelium, immune-rich areas containing plasma cells, and LAs (Fig. 7c–f). These LAs were enriched in both ACD and TCD.

These lamina propria LAs were located adjacent to stem cell niches and muscularis mucosa (Fig. 7g–i), with enrichment of T_FH_-like CD4^+^ T cell, T_reg_ cell and B cell gene signatures. Plasma cell signatures were more widely dispersed in immune-rich regions (Fig. 7i–k and Extended Data Fig. 9d,e). Genes for chemokines and receptors, including *CXCR5*, *CCR7*, *CCL19*, *CCL21*, *CXCL13* and *CXCL14* were enriched specifically within LAs (Fig. 7l), as were genes associated with S3 stromal cells, and pro-inflammatory stroma seen in inflammatory bowel disease^52^ (Fig. 7m).

### Receptor–ligand and GWAS candidate gene expression in CD

**Fig. 8:**
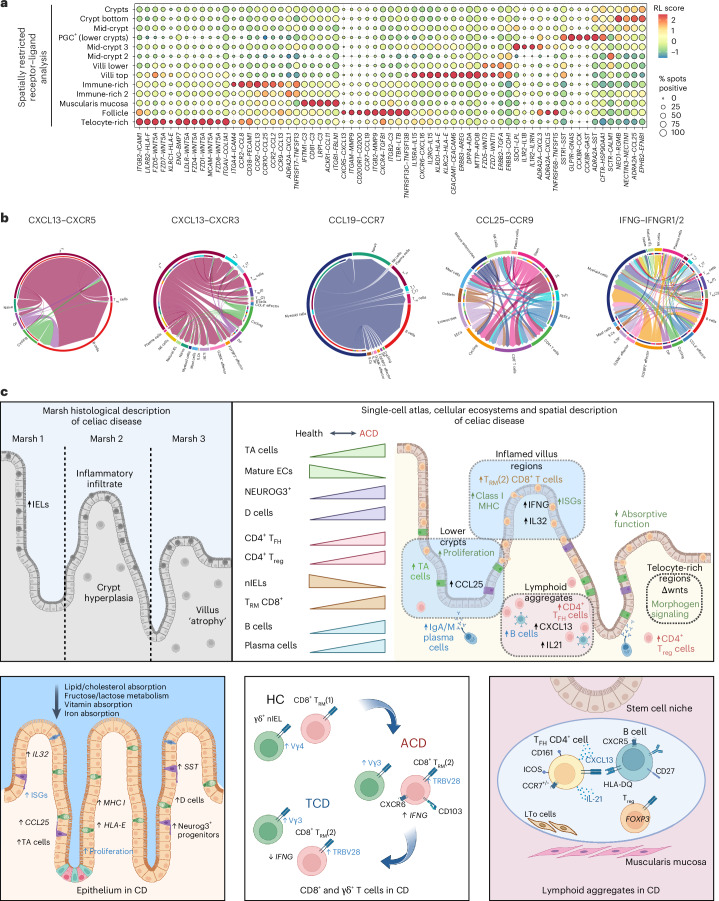
A spatially resolved model of mucosal immunological responses in CD. **a**, Bubble plot of region-specific receptor–ligand expression within the duodenal mucosa. Scaled receptor–ligand (RL) expression indicated by color; proportion of regions expressing the receptor–ligand genes indicated by bubble size. **b**, Circos plots of selected receptor–ligand pair expression between cell types in CD (dataset 1). **c**, A proposed schematic for the spatially resolved cellular ecosystems within the duodenal mucosa in CD. LTo, lymphoid tissue organizer. Figure created with BioRender.com.

We examined the expression of chemokines, cytokines and tumor necrosis factor (TNF) superfamily members, as well as receptor–ligand coexpression, within regions in the spatial transcriptomics dataset (Fig. 8a,b and Supplementary Fig. 8). Signaling pathway expression was region dependent, indicating highly localized mucosal signaling circuits (Fig. 8a). Within CD-specific LAs, chemokine signaling circuits involving *CXCR5–CXCL13*, *CCR7–CCL19*, *CXCR4* and integrins *ITGB2* and *ITGAM* were upregulated (Fig. 8a and Supplementary Fig. 8a,b). TNF superfamily receptor–ligand pathways were upregulated in LAs, including *TNF*, lymphotoxins A and B, *CD40*, *TNFRSF8* (CD30), *TNFRSF6B*, *TNFRSF18* (GITR) and *TNFRSF4* (OX40) (Supplementary Fig. 8c,d). There was also evidence of *IL2* and *IL21* signaling, as well as possible involvement of *IL23A* and *IL26* pathways (Supplementary Fig. 9e,f). LAs found in CD were enriched with signaling pathways involving *CXCR4*, *CXCR5* and *CXCL13* (Figs. 7l and 8a,b). Single-cell examination of *CXCL13*, *CCR7* and *ITGB2* signaling interactions in dataset 1 implicated T_FH_-like CD4^+^ T cell, B cell and myeloid cell interactions as drivers of these signaling pathways in LAs (Fig. 8b).

Villus tip regions, demonstrated region-specific activation of T cell and immune-related pathways, including increased expression of *IL15*, *IL18*, *IFNG* and IL32, and interactions including *CXCR6–CXCL16*, *CCR9–CCL25*, *DPP4–ADA* and *HLA-E* interactions with *KLRC2* and *KLRD1* (Fig. 8a and Supplementary Fig. 8). There was also high expression of TNF superfamily members associated with apoptotic pathways, including *FASLG*, *TNFSF10* (TRAIL), *TNFSF11* (RANKL), *TNFSF12* (TWEAK) and *TNFSF13* (APRIL) interactions. Examination of single-cell signaling pathways implicated CD8^+^ T cells in chemokine and type II interferon signaling, including *CCR9*–*CCL25* axis interactions between progenitor ECs and cycling CD8^+^ T cells (Fig. 8b). Wnt signaling pathways were enriched in telocyte-rich areas and neighboring villus structures (Fig. 8a). Such morphogen gradients may shape villus structure and morphology, perhaps driven by subepithelial telocytes^53^.

**Extended Data Fig. 10:**
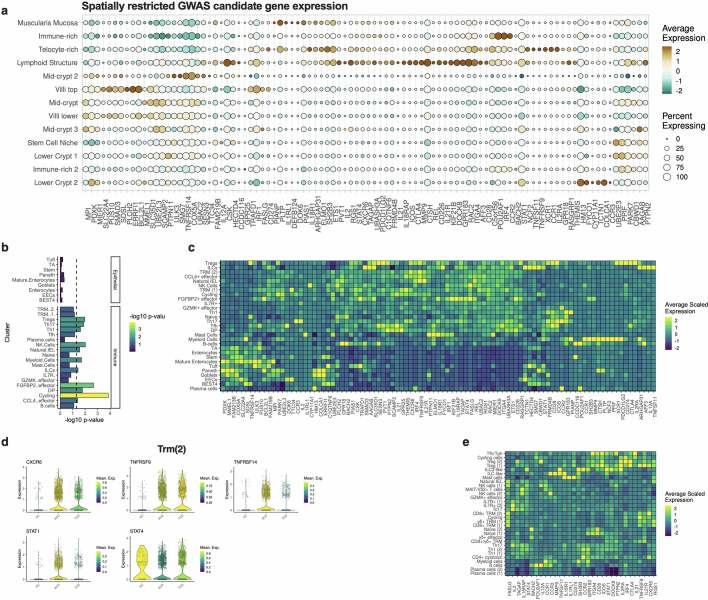
CD-related GWAS candidate genes show spatial region and cell-type specific expression. (**a**) Bubble plot of CD-associated GWAS candidate gene expression within the duodenal mucosa. Scaled gene expression indicated by colour, proportion of regions expressing the genes indicated by bubble size. (Genes identified from van der Graff et al. 2021, with at least one line of supporting evidence for direct gene association with CD). (**b**) Barplot of significance (–log10(FDR)) of CD-specific GWAS signal enrichment in scRNA-seq cell clusters in immune and epithelial subpopulations in cells from healthy and UC donors (n = 8). SNPsea empirical distribution *P* value with multiple testing correction (Benjamini–Hochberg). (**c**) Heatmap of CD-associated GWAS candidate gene scaled expression within immune and epithelial cell populations identified in Dataset 1 scRNA-seq dataset. (**d**) Violin plots of selected GWAS candidate gene expression in T_RM_(2) CD8^+^ T-cells in health and CD. (**e**) Heatmap of CD-associated GWAS candidate gene scaled expression within immune cell population identified in Dataset 2 scRNA-seq dataset.

To understand how genetic susceptibility can drive CD inflammatory responses, we examined putative genome-wide association study (GWAS) candidate gene expression^4^ in spatial regions. Villus, telocyte-rich and LA regions showed enriched expression of multiple GWAS candidates (Extended Data Fig. 10a). Expression of *MMP9*, *CTLA4*, *ICOS*, *ITGA4*, *GPR183*, *IL21* and *IL21R* was enriched within CD-specific LA regions (Extended Data Figs. 9e and 10a), while *IL2RA*, *CCR1*, *XCR1*, *TNFSF11* (RANKL) and *TNFRSF9* (CD137, 4-1BB) were increased in telocyte-rich regions.

scRNA-seq data were examined for cell-type-specific expression of CD genetic susceptibility loci. GWAS candidate genes were most prominent in T cell subsets (Extended Data Fig. 10b), with the highest signal enrichment in cycling CD8^+^ T cells. Specific putative GWAS candidate genes drove these associations (Extended Data Fig. 10c), with T_FH_-like CD4^+^ T cells expressing the *IL21*, *PTPN2*, *ITGA4*, *CD28* and *ICOS* and T_RM_(2) CD8^+^ T cells expressing *CXCR6*, *TNFRSF9* and *TNFRSF14*, and showing CD-related changes in *STAT* expression (Extended Data Fig. 10d). Cell-type-specific gene expression patterns were recapitulated in dataset 2 (Extended Data Fig. 10e).

## Discussion

This multi-omics study provides an integrated single-cell transcriptomic and proteomic assessment of intestinal immune, epithelial and parenchymal cell populations in adult and pediatric CD, contextualized through integration with spatial transcriptomics analysis. Our results show that perturbations of immune and epithelial cell states are spatially localized within distinct mucosal niches. Disease-associated cell types, including gluten-specific T_FH_-like CD4^+^ T cells and CD8^+^ T_RM_ cells, occupy distinct LA and villus niches, respectively, with cell–cell interactions best understood through spatial colocalization (Fig. 8c). The application of receptor–ligand analyses implicates broader cytokine and chemokine perturbations in CD than those described previously, including IL-32, CXCL13, CCL19, CXCL16, CXCL8 and CCL25.

Our understanding of human duodenal lymphoid structures is incomplete^54^. Isolated gut lymphoid structures may act as immune-inductive sites^55^. In CD, it remains unclear where the immune response to gluten is primed, or where subsequent antigen presentation occurs. Both myeloid and B cell lineages have been posited as relevant antigen-presenting cells (APCs) in CD^14^. The discovery of highly localized LAs where B cells and (likely gluten-specific) T_FH_ CD4^+^ T cells are co-located implicates these sites in gluten peptide antigen presentation.

Several aspects of the mucosal epithelial and immune response remained perturbed despite a GFD. These findings help explain the observation that subtle abnormalities in duodenal biopsy samples remain despite treatment, with reduced villus height/crypt depth ratios on morphometric analysis^2,56^. Whether this represents subclinical inflammation from ongoing low-level antigen exposure, slow mucosal healing or a long-term (perhaps epigenetic) response to prior inflammation is unclear. Participants often report ongoing symptoms despite a GFD, and this epithelial and immunological ‘scar’ from prior inflammation could underpin this, representing a therapeutic target.

Intestinal tissue-resident CD8^+^ T cell perturbations, including increases in T_RM_(2) populations, persisted despite GFD treatment. Therefore, this CD8^+^ T_RM_(2) state may represent a distinct T cell fate, rather than an activated phenotype alone. T_RM_ cells are long-lived memory populations that persist as immunological sentinels in barrier tissues, and this result is consistent with prior work showing a permanent reshaping of γδT cell-resident populations following CD-driven inflammation^51^.

CD8^+^ T cell populations exhibited changes in TCR repertoire, a finding validated in multiple datasets. These TCR repertoire changes, along with upregulation of TCR signaling gene sets, may indicate that TCR-dependent activation is relevant in CD, involving a separate mechanism to previously described NKG2C/NKG2D pathways^17,18^, and invoke the possibility of TCR-targeted disease therapies^57^. IL-15 and NK cell receptor signaling could lead to a reduction in TCR activation threshold, which could enable recognition of low-affinity antigens, either self-antigens or those of microbial or dietary origin^58,59^. The persistence of CD8^+^ T_RM_(2) cells in TCD could represent ongoing antigen exposure.

There is a series of enterocyte transcriptional states in the human small intestine, with absorptive cellular machinery generally limited to mature ECs, consistent with murine studies^60^. The shift to progenitor states in CD may increase CCL25 expression, implicating the CCL25–CCR9 axis in disease. This shift toward progenitor states underpins CD-associated malabsorption, beyond reduction in intestinal surface area.

In contrast to the term ‘villus atrophy’, we observe that the CD epithelium is hyperproliferative, and so the loss of villus structures requires additional explanation. Spatial transcriptomics data indicate specific regions responsible for WNT signaling, and we hypothesize that CD inflammation drives morphogen signaling shifts causing mucosal remodeling. These morphogen responses may not be CD specific, and may underpin histological similarities seen with mimics such as environmental enteropathy, monogenic enteropathies and olmesartan enteropathy^61^.

Integrating single-cell and spatial transcriptomics data, we have dissected the molecular and cellular basis of the histological changes in CD, including villus epithelial changes, crypt hyperplasia and intraepithelial lymphocytosis. This cellular, spatial transcriptomics description builds on the Marsh–Oberhuber histological description, with complex, highly localized, mucosal cell communities, including focal lymphoid organization where specific cell types, including gluten-specific T_FH_-like CD4^+^ T cells, B cells and T_reg_ cells, are co-located. Overall, our study of the mucosal cellular and spatial landscape in CD provides a detailed foundation from which to explore potential therapeutic targets, and highlights the need to explore the clinical implications of the prolonged epithelial–immune scar in TCD.

## Methods

### Human study participant recruitment and ethics

Study participants with CD, and HCs, were identified via Oxford University Hospitals NHS Trust CD clinic and endoscopy service (Oxford, UK). Blood and intestinal biopsy samples were taken at endoscopy with informed consent under the Oxford Gastrointestinal Illnesses Biobank study (REC: 21/YH/0206). Study participant demographics and study inclusion/exclusion criteria are summarized in Supplementary Table 1. Participants were not compensated financially.

### Peripheral blood mononuclear cell isolation

Peripheral blood mononuclear cells were extracted from whole blood or leukocyte cones via density gradient centrifugation. Briefly, peripheral blood was diluted at a 1:1 ratio with Dulbecco’s phosphate buffered saline without calcium or magnesium (PBS) and layered over Lymphoprep (Axis-Shield) before centrifugation (973*g* for 30 min at 20 °C without brake). The mononuclear layer was retrieved and washed twice in PBS or R10 culture media (RPMI-1640 (Sigma-Aldrich), 10% FCS, 1% penicillin/streptomycin, 1% l-glutamine). Viable mononuclear cells stained with Trypan blue were counted manually by microscopy using a hemocytometer before downstream applications. If remnant red blood cells were present, they were lysed with ammonium–chloride–potassium solution for 2–3 min, then washed again in R10. Samples were cryopreserved in freezing medium (90% FCS (Sigma-Aldrich), 10% dimethylsulfoxide (Sigma-Aldrich)). When needed, samples were thawed rapidly in a water bath (37 °C), then washed twice in R10 before downstream use.

### Intestinal biopsy collection, preservation and dissociation

Intestinal biopsy samples were collected at endoscopy from duodenum. Biopsy samples for intestinal lymphocyte extraction were immediately placed in sterile R10 medium (as above) or MACS Tissue Storage Solution (Miltenyi Biotec) on ice for transportation, before cryopreservation in CryoStor Cs10 (STEMCELL Technologies). This approach preserves immune cell viability and surface marker expression^62^. When required, samples were rapidly thawed in a 37 °C water bath and washed in 20 ml R10 before tissue dissociation.

For immune cell isolation from duodenal biopsy samples for scRNA-seq (10x Genomics, dataset 1), biopsy samples were incubated in R10 medium with 1 mg ml^−1^ Collagenase D (Roche) and 100 mg ml^−1^ DNase (Thermo Fisher Scientific) for 1 h in a shaking incubator at 37 °C. Biopsy samples were then dissociated by vigorous agitation using a GentleMACS Dissociator (Miltenyi Biotec), then strained through a 70-μm filter. The mononuclear cells were isolated on a discontinuous 70% and 35% Percoll gradient (GE Healthcare) by centrifugation at 700*g* for 20 min without brake. Mononuclear cells were collected from the interface and washed in R10. Cells were washed with R10 medium before antibody staining and downstream applications. The complete protocol with all steps is available at https://www.protocols.io/view/freezing-and-processing-intestinal-biopsies-for-th-dm6gp8745lzp/v1/ (Oxford HCA, 2019).

The method for EC isolation from duodenal biopsy samples for scRNA-seq (10x Genomics, dataset 1) was adapted from ref. ^35^. Biopsy samples were washed in wash medium (HPGA, 1 mM EDTA, 1 mM dithiothreitol), then incubated in chelation medium (HPGA, 1 mM EDTA) at 37 °C for 40 min with agitation. The supernatant, which was removed and replaced every 10 min and contained epithelial crypts, was digested into a single-cell suspension by dissociation in a shaking incubator with TrypLE Express and DNase (50 µg ml^−1^) for 60 min at 37 °C. The epithelial single-cell suspension was washed with PBS and passed through a 30-µm filter. Cell counts and viability were confirmed with a manual hemocytometer before further processing. The complete protocol with all steps is available at https://www.protocols.io/view/isolation-of-cells-from-the-epithelial-layer-of-fr-e6nvw9e6dgmk/v1/ (Oxford HCA, 2019).

For immune cell isolation from duodenal biopsy samples for scRNA-seq and proteomics (BD Rhapsody, dataset 2), samples were thawed, then diluted with warm X-VIVO (Lonza) + 1% AB serum (Sigma-Aldrich). Biopsy samples underwent enzymatic and mechanical digestion using 0.042 mg ml^−1^ Liberase TL (Roche) and 1 mg ml^−1^ DNAse I (Thermo Fisher). Samples were placed horizontally in a shaking incubator for 20 min at 37 °C and homogenized using a gentleMACS Dissociator (Miltenyi Biotech). Dissociated cells were passed through a 70-μm strainer. Lymphocytes were enriched by Percoll density centrifugation as above.

Duodenal biopsy samples for flow cytometry were processed as for scRNA-seq of immune cells (see above). Cells were washed with R10 medium before antibody staining and downstream applications.

Duodenal biopsy samples for fluorescence-activated cell sorting (FACS) of IELs for RNA-seq and TCR repertoire sequencing were placed in 10 ml HBSS with 1 mM EDTA and 1 mM dithiothreitol (both Sigma-Aldrich) and placed in a shaking incubator (200 rpm, 37 °C) for 15 min. IELs were strained through a 70-μm filter and washed two to three times with R10 before downstream applications.

For spatial transcriptomics, single intestinal biopsy samples were embedded in OCT cryo-embedding matrix (Thermo Fisher Scientific) then frozen in isopentane (Sigma-Aldrich) suspended over liquid nitrogen or dry ice, and stored at −80 °C until use.

### Flow cytometry and cell sorting

For surface marker staining, cells were stained in 50 ml of FACS buffer (PBS + 1 mM EDTA + 0.05% BSA) for 30 min at 4 °C. Surface antibodies and clones used are listed in Supplementary Table 3. Antibodies were purchased from BioLegend, BD Biosciences, Miltenyi Biotec or Thermo Fisher Scientific.

After staining, cells were stored at 4 °C protected from light until data acquisition. Flow cytometry data were acquired on a BD LSR II flow cytometer (BD Biosciences). FACS samples were surface stained as above, with Sytox Green (Thermo Fisher Scientific) used as a viability dye. FACS was performed on an Aria III (BD Biosciences; 70-mm nozzle).

For sorting by FACS for scRNA-seq of intestinal immune populations, cells were stained with EpCam-PE, CD27-BV421 and CD45-APC-Cy7. Live CD45^+^ or CD27^+^ cells were sorted to include all mucosal immune cell populations, including long-lived CD27^+^ plasma cells, which can downregulate surface CD45 expression.

For sorting by FACS for scRNA-seq of intestinal epithelial populations, cells were stained with EpCAM-PE and CD45-AF700, with live EpCAM^+^ cells sorted.

For sorting by FACS for bulk RNA-seq or TCR-seq of CD8^+^ IEL populations, cells were stained with CD45-BV785, CD3-BV711, αβTCR-APC, γδTCR-PE, CD4-BV650 and CD8a-AF700, with live CD45^+^CD3^+^αβTCR^+^CD8^+^CD4^−^ cells sorted.

### Single-cell RNA-seq (10x Genomics)

Libraries were generated using 10x Genomics Chromium Single Cell V(D)J Reagents Kits (v1 Chemistry) per the manufacturer’s instructions. Sorted cells suspended in PBS (plus 0.04% BSA) at a concentration of 1,000 cells per microliter were loaded into one lane of a Chromium controller. Library quality and quantity were assessed using a TapeStation (Agilent) and Qubit Fluorometer (Thermo Fisher Scientific). Libraries were sequenced on an Illumina HiSeq 4000 following the manufacturer’s instructions. Library generation and sequencing were performed at the Sanger Institute, Cambridge.

### Targeted single-cell RNA-seq and AbSeq (BD Rhapsody)

Sorted CD45^+^ cells were stained with a cocktail of 79 oligonucleotide-conjugated AbSeq antibodies (BD Biosciences, for 45 min at 4 °C. Cells were then washed to remove residual unbound AbSeq antibodies and loaded onto three BD Rhapsody cartridges (BD Biosciences) for single-cell capture. AbSeq antibodies used in this study are listed in Supplementary Table 2.

Single-cell capture and cDNA library preparation were performed using the BD Rhapsody Express Single-Cell Analysis System (BD Biosciences), according to the manufacturer’s instructions. Briefly, cDNA was amplified—ten cycles for resting cells and nine cycles for in vitro-stimulated cells—using the Human Immune Response Primer Panel (BD Biosciences; Supplementary Table 6), containing 399 primer pairs and a supplementary panel of 105 primer pairs (BD Biosciences; Supplementary Table 3).

The resulting PCR1 products were purified using AMPure XP magnetic beads (Beckman Coulter), and the respective mRNA and AbSeq/Sample Tag products were separated based on size selection, using different bead ratios (0.7× and 1.2×, respectively). The purified mRNA and Sample Tag PCR1 products were further amplified (ten cycles), and the resulting PCR2 products purified by size selection (1× and 1.2× for the mRNA and Sample Tag libraries, respectively). The concentration, size and integrity of the resulting PCR products were assessed using both Qubit (High-Sensitivity dsDNA Kit; Thermo Fisher) and the Agilent 4200 TapeStation system (High Sensitivity D1000 ScreenTape; Agilent). The final products were normalized to 2.5 ng μl^−1^ (mRNA), 0.5 ng μl^−1^ (Sample Tag) and 0.275 ng μl^−1^ (AbSeq) and underwent a final round of amplification (six cycles for mRNA and eight cycles for Sample Tag and AbSeq) using indexes for Illumina sequencing to prepare the final libraries. Final libraries were quantified using Qubit and Agilent TapeStation and pooled (~60%/38%/2% mRNA/AbSeq/Sample Tag ratios, respectively) to achieve a final concentration of 5 nM. Final pooled libraries were spiked with 10% PhiX control DNA to increase sequence complexity and sequenced (75 base pairs (bp), paired-end) on a HiSeq 4000 sequencer (Illumina).

### Spatial transcriptomics exploratory experiment (dataset 4)

Cryopreserved, OCT-embedded duodenal biopsy samples were stored at −80 °C until use. Before performing the full protocol, a tissue permeabilization optimization was performed (10x Genomics, Visium Spatial Tissue Optimization), which identified 11 min as the optimum permeabilization time.

Samples were processed for spatial transcriptomics per the manufacturer’s instructions (10x Genomics, Visium Spatial), with 2 × 10-μm sections cut on a pre-cooled cryostat for each sample onto two 6.5 × 6.5-mm capture areas, each with approximately 5,000 oligonucleotide-barcoded 55-μm-diameter spots. Slides were fixed, H&E stained and imaged on a Leica DMI8 Widefield microscope at a magnification of ×40. Tissue was permeabilized per instructions for 11 min, followed by reverse transcription and second-strand synthesis performed on the slide. cDNA quantification was performed using qPCR using KAPA SYBR FAST-qPCR kit (KAPA Biosystems) on a CFX96 Thermal Cycler instrument (Bio-Rad). Following library construction per instructions, the spatial transcriptomics libraries were quantified and pooled at a concentration of 4 nM with a sample ratio corresponding to the approximate surface area of tissue coverage obtained from the H&E imaging. Pooled libraries were sequenced on a NextSeq (Illumina) using a 150-bp paired-end dual-indexed setup (High output, v2.5, Illumina) loaded at a concentration of 1.8 pM, and sequenced to a manufacturer-recommended depth of a minimum of 50,000 reads per tissue-covered spot.

### ST validation experiment (dataset 5)

Cryopreserved, OCT-embedded duodenal biopsy samples were stored at −80 °C until use. Spatial transcriptomics was performed using the Visium CytAssist (10x Genomics) workflow for fresh-frozen tissue according to the manufacturer’s instructions. Ten-micron sections were cut on a cryostat pre-cooled to −20 °C and placed on 11 × 11-mm areas (four sections per area) on SuperFrost Plus slides (Thermo Fisher). Sections were fixed, H&E stained and imaged on an Axioscan Z1 slide scanner (Zeiss) at a magnification of ×20. Sections were de-stained and subjected to on-slide probe hybridization and ligation followed by probe transfer onto Visium CytAssist Spatial Gene Expression slides, each containing an 11 × 11-mm capture area covered by approximately 14,000 55-µm-diameter oligonucleotide-barcoded spots. Probes were extended and cDNA quantified by qPCR using a KAPA SYBR FAST-qPCR kit (KAPA Biosystems) on a CFX96 Thermal Cycler instrument (Bio-Rad), followed by off-slide library construction per the instructions. Libraries were quantified and pooled at a concentration of 2 nM with a sample ratio corresponding to the approximate surface area of tissue coverage obtained from the H&E imaging. Pooled libraries were sequenced on a NextSeq 500 instrument (Illumina) using a 150-bp paired-end dual-indexed setup (High output, v2.5, Illumina) at a manufacturer-recommended depth of a minimum of 50,000 reads per tissue-covered spot.

### Bulk RNA and TCR repertoire sequencing

A TRIzol nucleic acid extraction method was used to extract RNA from low numbers of sorted lymphocytes, as previously described^63,64^, except that phase-lock gel tubes were replaced with standard 1.5 ml microcentrifuge tubes. Briefly, after sorting, cells were centrifuged (500*g*, 5 min), resuspended in 1 ml TRIzol, then frozen at −80 °C until RNA extraction. For RNA extraction, samples were thawed, mixed with 200 μl chloroform and centrifuged (14,000*g*, 5 min). A total of 500 μl of the aqueous phase was taken and RNA was extracted using the Agencourt RNAdvance Tissue Isolation kit. RNA concentration and purity were assessed on a 2100 Bioanalyzer instrument (Agilent).

Bulk RNA-seq was performed using the Smart-seq2 protocol^65^ at the Oxford Genomics Centre (University of Oxford). Around 10 ng RNA was used as a template from each sample for library generation. Barcoded samples were pooled, and External RNA Controls Consortium RNA (1:100,000 dilution) was added before 75-bp paired-end sequencing on an Illumina HiSeq 4000 instrument.

Bulk TCR repertoire sequencing was performed using the amplicon-rescued multiplex-PCR method (iRepertoire). This method performs an initial first-round RT–PCR with TCR V and C gene-specific primers for the relevant TCR chain, followed by further amplification steps with universal primers for the exponential phase of amplification. This method is designed to provide quantitative, deep sequencing of the TCR repertoire, with minimal bias. Library generation was performed following the manufacturer’s instructions, except for using 96-well plates. The quality, size distribution, concentration and presence of contaminating primer dimers of the final product was assessed using agarose gel electrophoresis, a spectral photometer (Nanodrop, Thermo Fisher Scientific), and the Bioanalyser DNA 1000 assay using a 2100 Bioanalyzer instrument (Agilent). Libraries were quantified using the KAPA Library Quantification Kit (Roche) on a CFX96 Thermal Cycler instrument (Bio-Rad) before equimolar pooling. A PhiX library spike-in was added (10%) due to the low diversity of the TCR library, before 300-bp paired-end sequencing on an Illumina MiSeq instrument at the Oxford Genomics Centre.

### Quantification, statistical and computational analysis

#### Statistics and reproducibility

This was an observational, descriptive study. The experiments were not randomized. No statistical method was used to predetermine sample size, but our sample sizes are similar to those reported in previous publications^27,31,35^. No data were excluded from analyses. The investigators were not blinded to allocation during experiments and outcome assessment. Data collection and analysis were not performed blind to the conditions of the experiments. Data distributions of transcriptomics datasets were tested to ensure they met the assumptions of statistical tests. For other datasets, data distribution was assumed to be normal, but this was not formally tested.

#### Graphs, statistics and flow cytometry analysis

All statistical analyses and graphs, except transcriptional data, were performed using Prism Software v9 and v10 (GraphPad). Specific statistical tests are described in relevant figure legends. All data are presented as the mean ± s.e.m. unless stated otherwise. Flow cytometry data were analyzed using FlowJo v9.9.5 and v10.6.1.

### Computational data analyses

#### Bulk RNA-seq analyses

Raw sequencing read data were subjected to quality control and aligned to the human reference hg38 genome using STAR aligner. The DESeq2 R package was used for downstream differential expression analysis.

#### Bulk TCR repertoire analysis

Bulk TCR repertoire analysis was performed using the iRepertoire analysis pipeline.

#### scRNA-seq data analysis

Raw read data were processed with either the Cell Ranger pipeline or the BD Genomics pipeline. Downstream analyses were carried out in R using the Seurat pipeline.

#### Spatial transcriptomics data analysis

Raw read data were processed using the Space Ranger pipeline. Downstream analyses were carried out in R using the Seurat pipeline.

#### Single-cell TCR analysis

Raw sequencing read data were processed with the Cell Ranger VDJ pipeline. Downstream analyses were carried out in R.

Additional details for computational data analysis are provided in Supplementary Methods.

### Reporting summary

Further information on research design is available in the Nature Portfolio Reporting Summary linked to this article.

## Online content

Any methods, additional references, Nature Portfolio reporting summaries, source data, extended data, supplementary information, acknowledgements, peer review information; details of author contributions and competing interests; and statements of data and code availability are available at 10.1038/s41590-025-02146-2.

## Supplementary information

Supplementary Information Supplementary Figs. 1–8, Methods, References and description of tables.

Reporting Summary

Supplementary Tables 1–7 Supplementary Table 1. Study participant demographics and clinical information. Supplementary Table 2. BD Rhapsody gene expression probes. Supplementary Table 3. BD Rhapsody AbSeq panel. Supplementary Table 4. Description of epithelial cell clusters. Supplementary Table 5 Differential expression of Gene Ontology terms in epithelium (active CD versus controls). Supplementary Table 6. Description of CD4^+^ T cell clusters. Supplementary Table 7. Description of CD8^+^ T cell clusters

## Data Availability

Raw and processed data are available on Zenodo (10.5281/zenodo.15069144 (ref. ^66^)) and Gene Expression Omnibus (GSE252545).
