# Supplementary material for: Immune–epithelial–stromal networks define the cellular ecosystem of the small intestine in celiac disease
Source: Nat Immunol. 2025 May 6;26(6):947–62. doi: 10.1038/s41590-025-02146-2 (PMC12133578; doi:10.1038/s41590-025-02146-2)
Supplement: Supplementary file 1 — Supplementary Figs. 1–8, Methods, References and description of tables. [file 41590_2025_2146_MOESM1_ESM.pdf]

nature immunology

Supplementary information https://doi.org/10.1038/s41590-025-02146-2

Immune–epithelial–stromal networks define the cellular ecosystem of the small intestine in celiac disease

In the format provided by the authors and unedited

Supplementary Figure 1

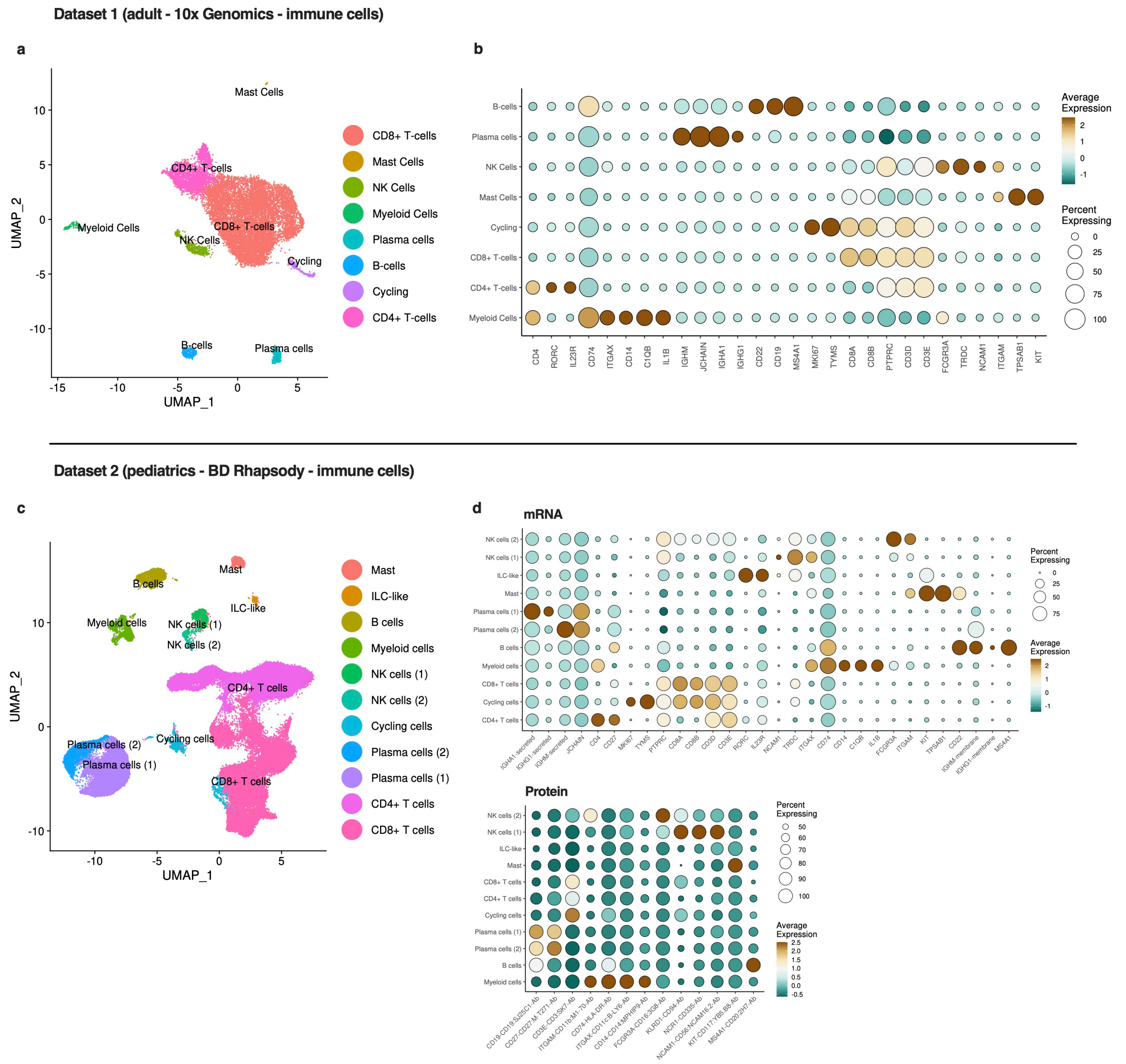

Supplementary Figure 1: Single-cell RNA sequencing (scRNA-seq) of immune populations in celiac disease (CD)

Dataset 1 CD45⁺ small intestinal immune cells in health and CD (a-b). (a) UMAP plot of small intestinal immune cells in health (n=3) and CD (n=5). (b) Bubble plot showing the expression of selected genes defining specific cluster identities. Gene expression indicated by colour, proportion of cells expressing the gene indicated by bubble size. Dataset 2 CD45⁺ small intestinal immune cells in health and CD (c-d). (c) UMAP plot of small intestinal immune cells in health (n=5) and CD (n=10). (d) Bubble plot showing the expression of selected genes and surface proteins defining specific cluster identities. Gene/protein expression indicated by colour, proportion of cells expressing the gene indicated by bubble size.

Supplementary Figure 2

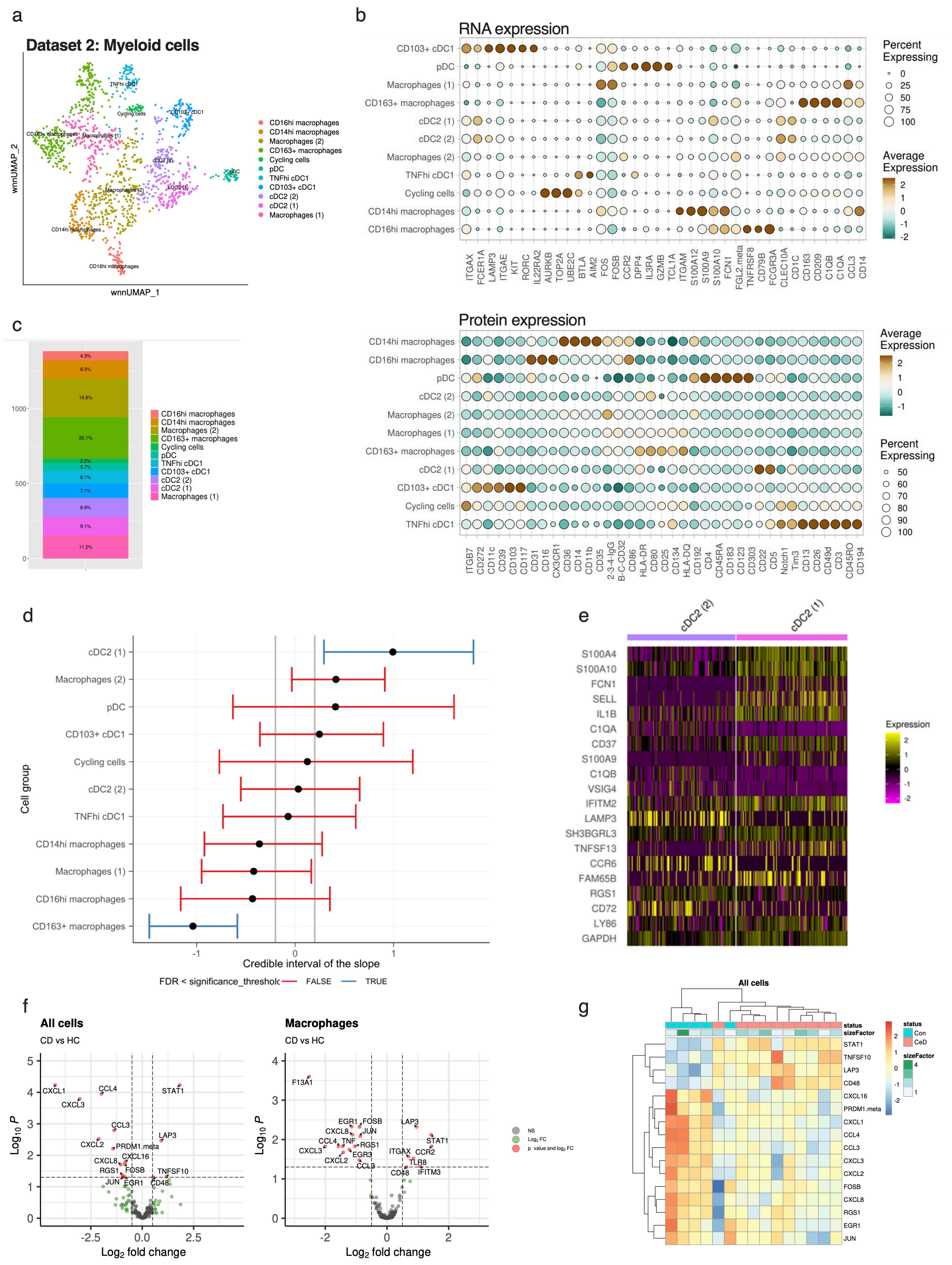

Supplementary Figure 2: Myeloid cells

Myeloid cell clusters were examined in the Dataset 2 (pediatric) dataset (BD Rhapsody) in healthy controls (HC) (n=5) and CD (n=10). (a) UMAP plot of intestinal myeloid clusters. (b) Bubble plot showing the expression of selected genes (above) and surface proteins (below) defining specific cluster identities. Gene/protein expression indicated by colour, proportion of cells expressing the gene indicated by bubble size. (c) Intestinal myeloid subset proportions. (d) Intestinal myeloid subset enrichment in CD vs HC (95% confidence intervals shown). (e) Heatmap of differentially expressed genes between conventional dendritic cell subtypes. (f) Volcano plots of differentially expressed genes between CD and HC in all myeloid cells (left) and macrophages (right). (g)Heatmap of differentially expressed genes between CD and HC in myeloid cells.

Supplementary Figure 3

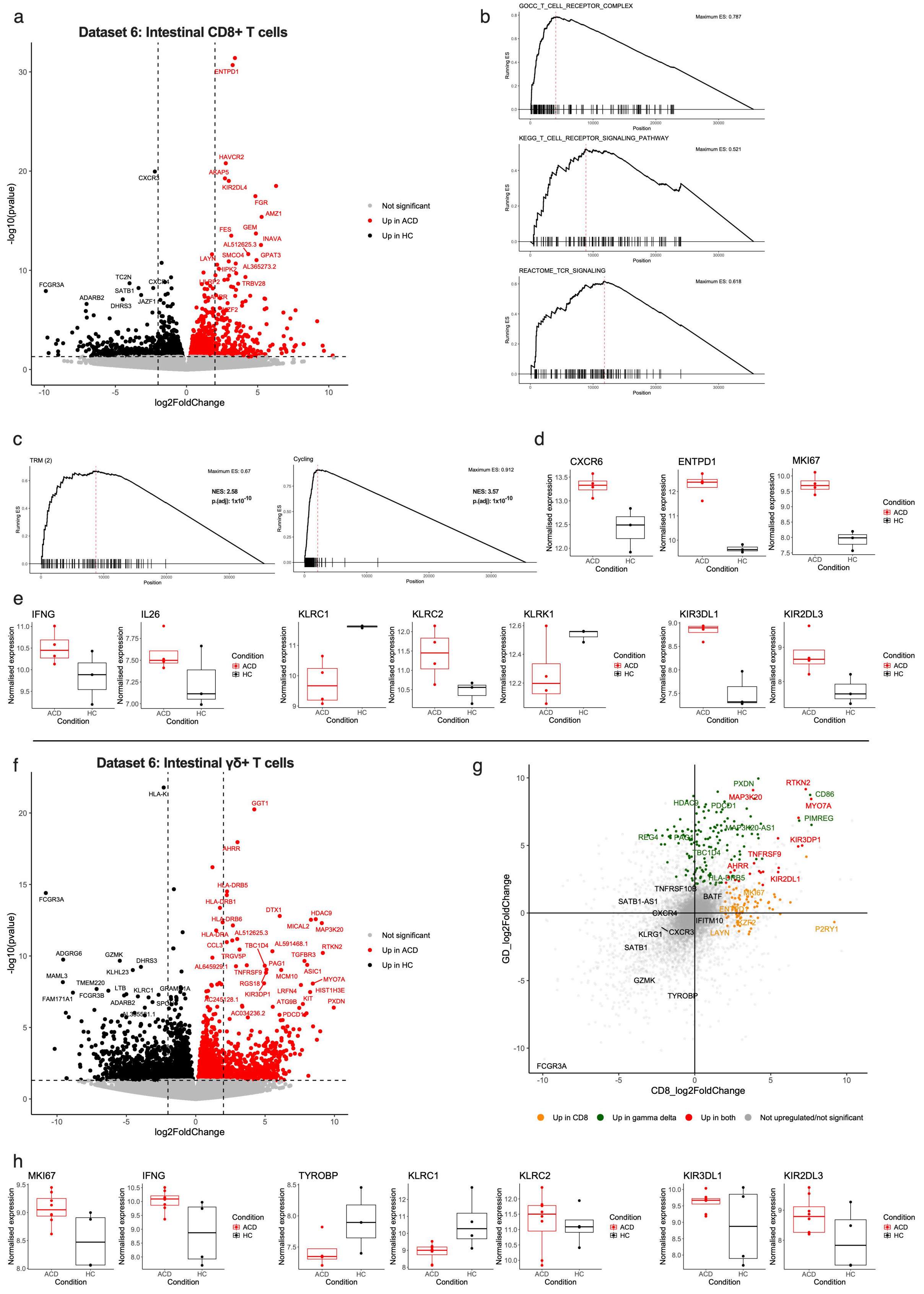

Supplementary Figure 3: Bulk RNA sequencing of CD8+ and γδ+ intestinal T cells in celiac disease

Bulk RNA sequencing of sorted CD8+ and γδ+ intestinal T-cells from subjects with CD (n=4) and healthy controls (n=3) was performed. (a) Volcano plot of differentially expressed genes between CD8+ T cells. (b) Gene set enrichment analysis (GSEA) of TCR activation gene sets. (c) Gene set enrichment analysis (GSEA) of CD8+ T cell subset gene signatures identified in single-cell experiments in the bulk RNA-seq dataset. (d) Expression of genes associated with T_{RM}(2) and cycling populations in CD8+ T cells. (e) Expression of cytokines, NK receptors, and KIRs in CD8+ T cells. (f) Volcano plot of differentially expressed genes between HC and ACD in γδ T cells (D). (g) Scatter plot of log2(Fold change) (log2FC) between ACD and HC samples for CD8+ T cells (x-axis) and γδ T cells (y-axis). Genes differentially expressed genes are identified by colour. (h) Expression of cytokines, NK receptors, and KIRs in γδ+ T cells in health and CD. (d,e,h) Median, IQR, range shown.

Supplementary Figure 4

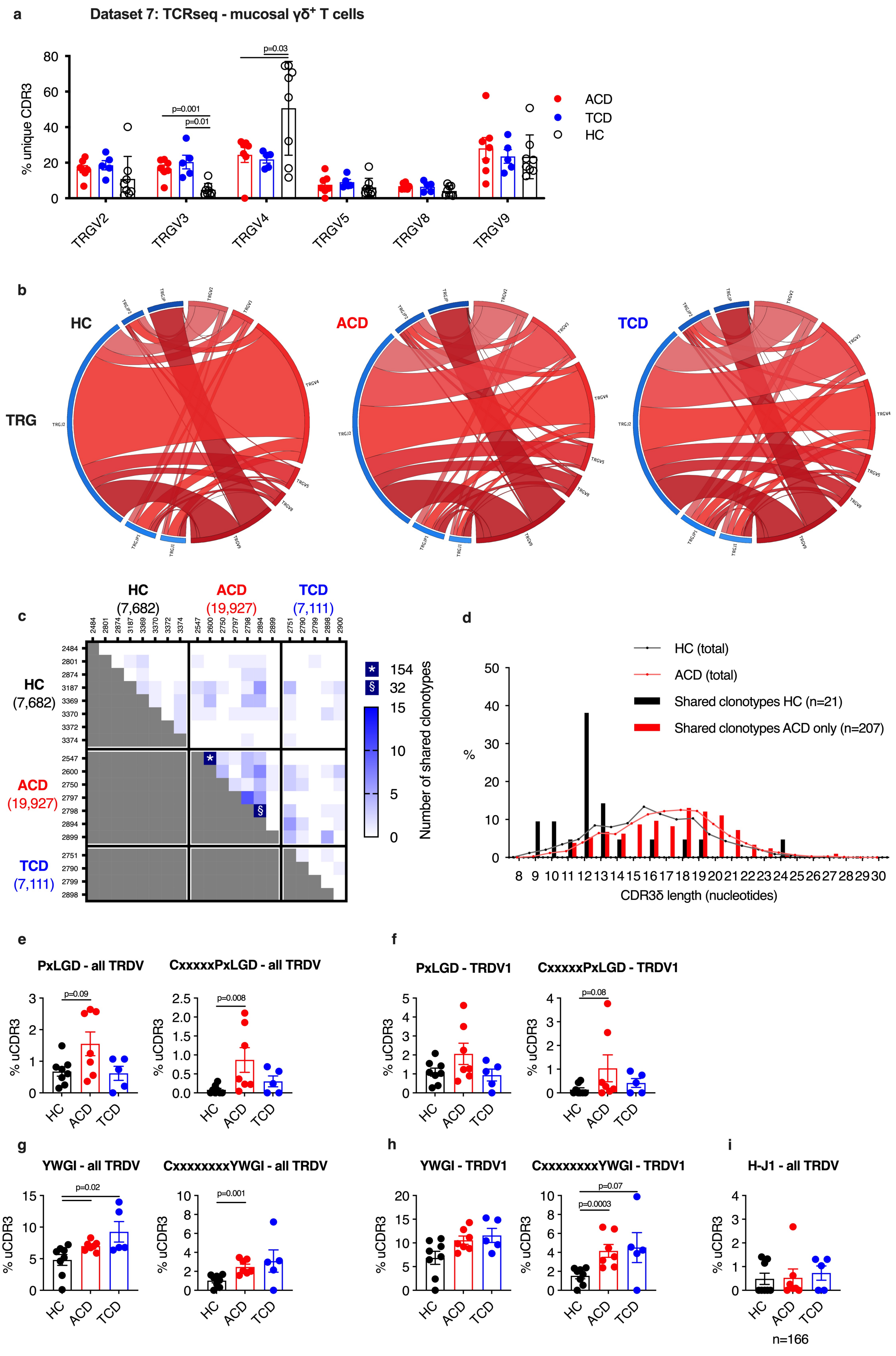

Supplementary Figure 4: TCR repertoire sequencing of intestinal γδ T-cells in celiac disease.

TCR repertoire sequencing of sorted intestinal γδ T-cells in healthy controls (HC, n=8), active CD (n=7); treated CD(n=5). (a) TRGV gene usage of unique CDR3 clonotypes. (b) Circos (chord) plots showing TRGV/TRGJ paired gene usage of unique CDR3 clonotypes. (c) Sharing of TRD clonotypes between study subjects with or without CD, displayed as a heatmap. (d) CDR3 length of total TRD clonotypes in health and active CD (black and red lines respectively, and CDR3 length of clonotypes shared between HC and ACD subjects (black and red bars respectively). (e-i) Frequency of reported putative celiac-related CDR3δ motifs in health and CD. (e-f) Frequency of the PxLGD CDR3δ motif in all TRDV intestinal clonotypes (e) or TRDV1 intestinal clonotypes only (f), either located anywhere in the CDR3 sequence (left), or defined by its position from the V-gene cysteine residue (right). (g-h) Frequency of the YWGI CDR3δ motif in all TRDV intestinal clonotypes (g) or TRDV1 intestinal clonotypes only (h), either located anywhere in the CDR3 sequence (left), or defined by its position from the V-gene cysteine residue (right). (i) Frequency of the H-J1 CDR3δ motif in all TRDV intestinal clonotypes. (a) 2-way ANOVA with Holm-Sidak's multiple comparisons test. (e-i) Two-tailed Mann-Whitney test. (a,e-i) Mean +/- SEM shown.

Supplementary Figure 5

Dataset 3: Stromal/endothelial scRNAseq

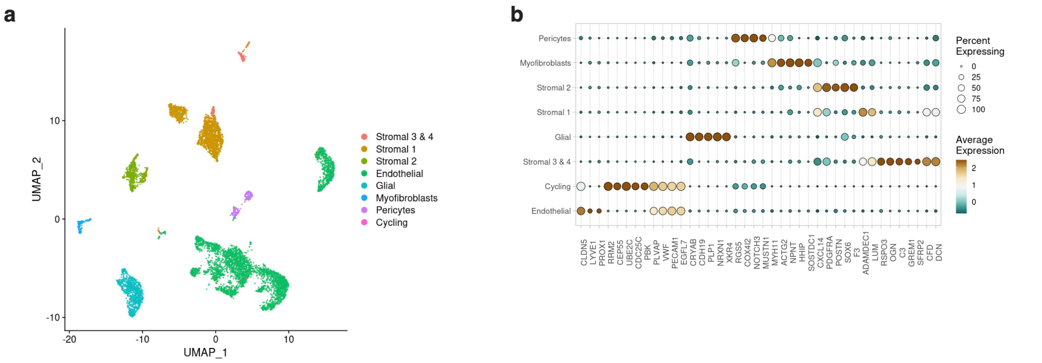

Stroma/fibroblast analysis (1517 cells)

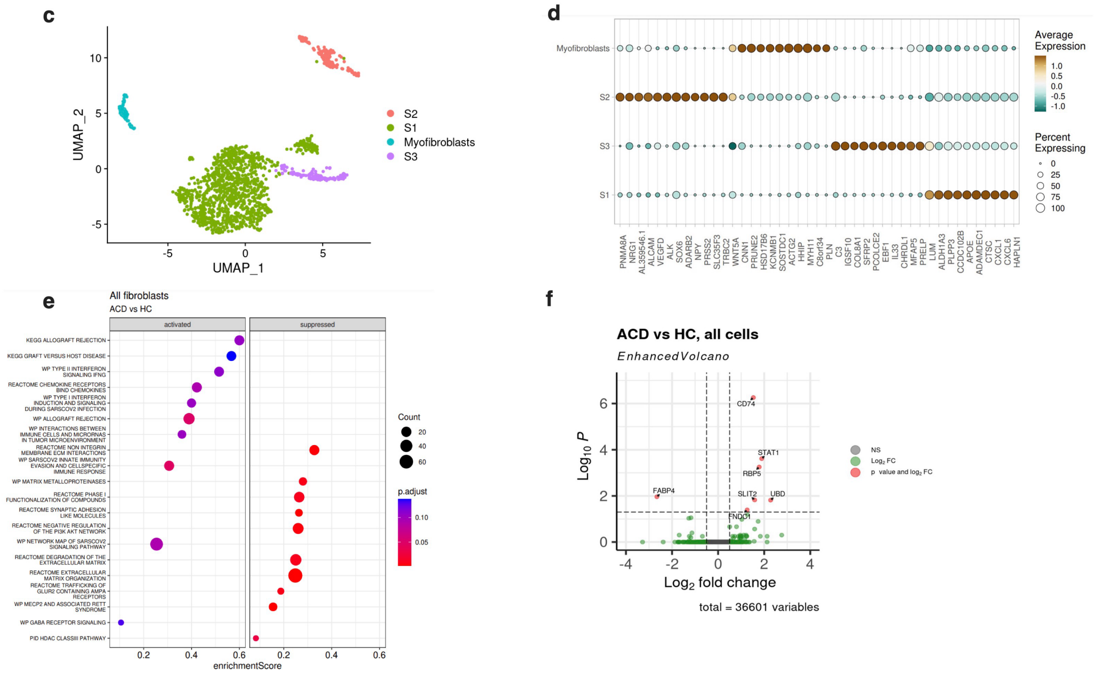

Endothelium analysis (2549 cells)

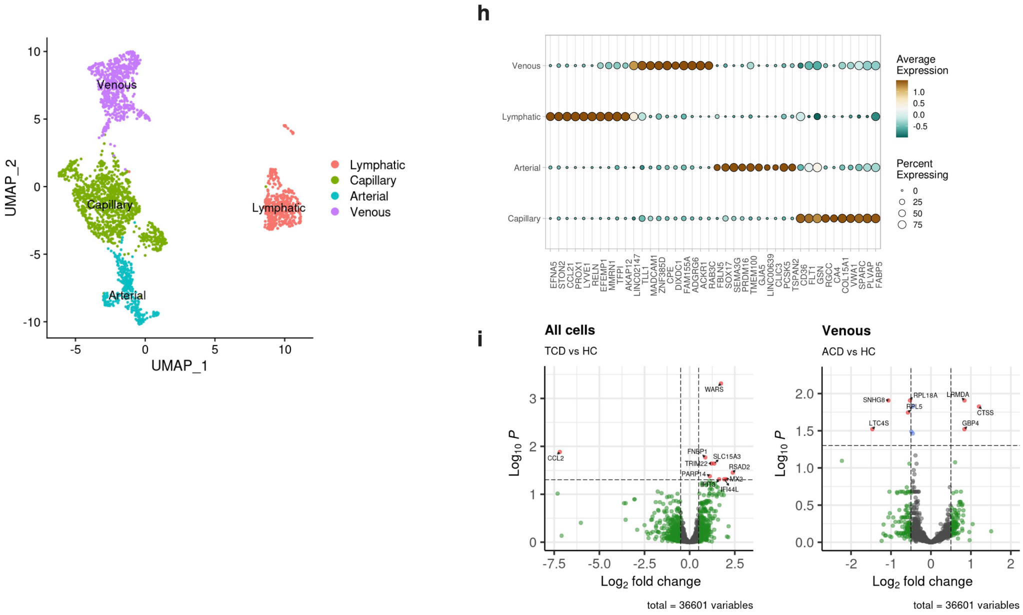

Supplementary Figure 5: Intestinal stromal and endothelial cell populations in CD

(a) UMAP plot of intestinal parenchymal cell populations in health, active and treated CD (n=12). (b) Bubble plot showing the expression of selected genes defining specific cluster identities. Scaled gene expression indicated by colour, proportion of cells expressing the gene indicated by bubble size. (c) UMAP plot of intestinal stromal and fibroblast populations in health, active and treated CD (n=12). (d) Bubble plot showing the expression of selected genes defining specific cluster identities. Scaled gene expression indicated by colour, proportion of cells expressing the gene indicated by bubble size. (e) GSEA analysis showing enrichment or reduction of pathways in stromal cells in active CD. (f) Volcano plot of differentially expressed genes in stromal cells between health and active CD. (g) UMAP plot of intestinal endothelial populations in health, active and treated CD (n=12). (h) Bubble plot showing the expression of selected genes defining specific cluster identities. Scaled gene expression indicated by colour, proportion of cells expressing the gene indicated by bubble size. (i) Volcano plot of differentially expressed genes in endothelial cells between health and treated CD (left), and in lymphatic endothelial cells between health and active CD (right).

Supplementary Figure 6

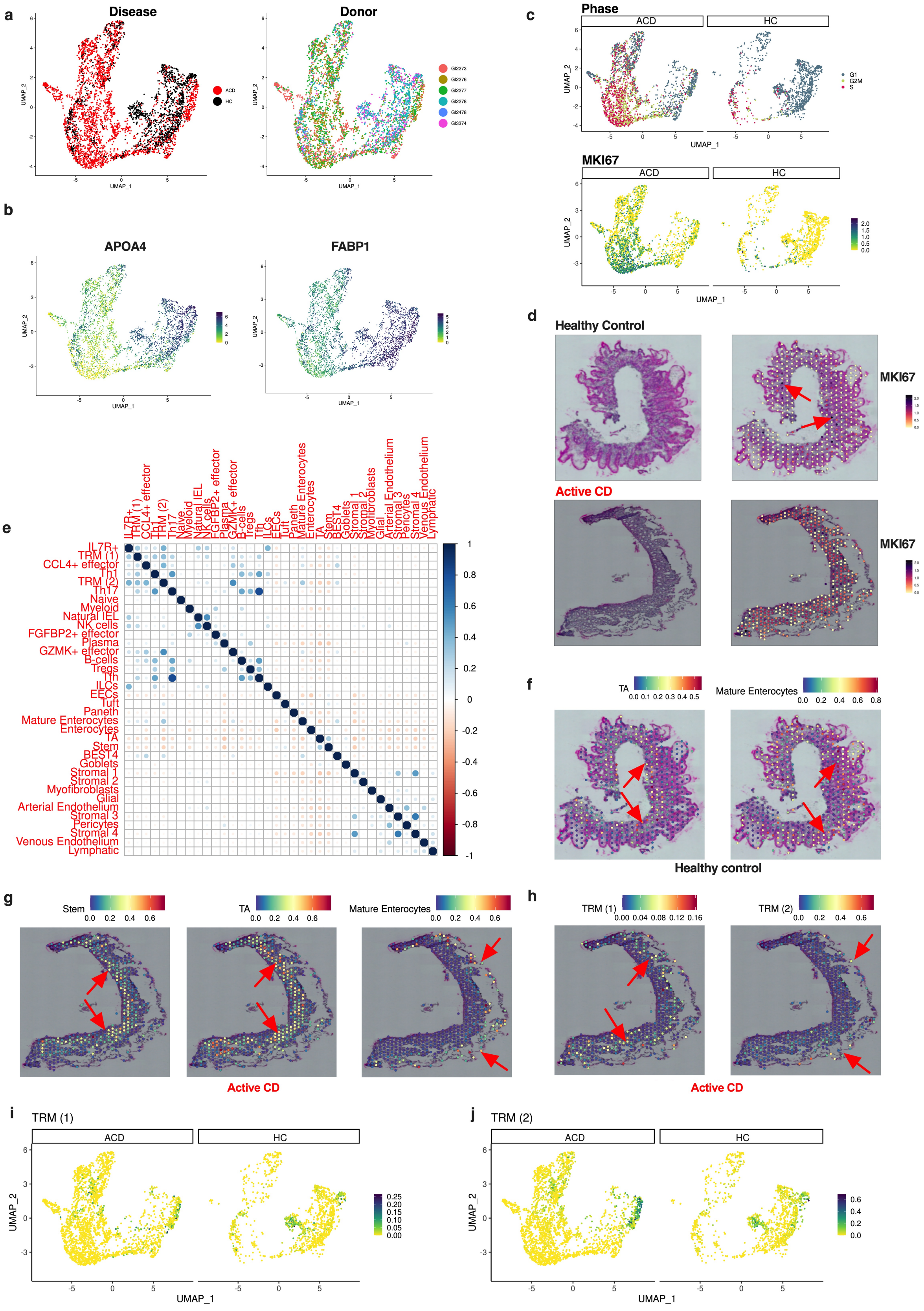

Supplementary Figure 6: Spatial Transcriptomics (1)

(a) UMAP plot of spatial transcriptomics tissue-covered spots, coloured by disease state (left) and study subject (right). (b) UMAP plot overlaid with expression if genes associated with enterocyte absorptive function. (c) UMAP overlaid with predicted cell cycle gene expression state (above) and expression of *MKI67* (below), split by disease state. (d) MKI67 expression on tissue spots of representative healthy control (above) and active CD (below) sample. (e) Correlation plot showing colocalization of predicted cell types in spatial transcriptomic data in active CD. (f) Predicted transit amplifying (TA) cell and mature enterocyte signatures in representative control sample. (g) Predicted stem cell, TA cell and mature enterocyte signatures in representative active CD sample. (h) Predicted $CD8^+$ $T_{RM}(1)$ and $T_{RM}(2)$ cell signatures in representative active CD sample. (i-j) UMAP plot overlaid with $T_{RM}(1)$ $CD8^+$ T-cell (i) and $T_{RM}(2)$ $CD8^+$ T-cell (j) signatures, split by disease state.

Supplementary Figure 7

a

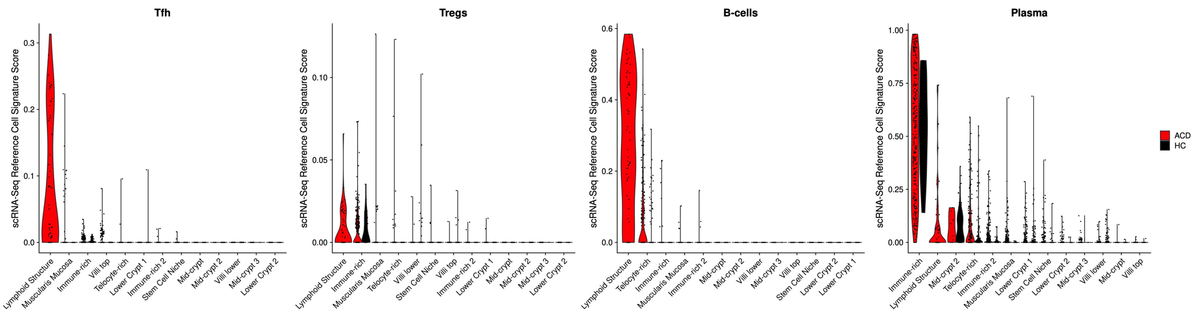

b

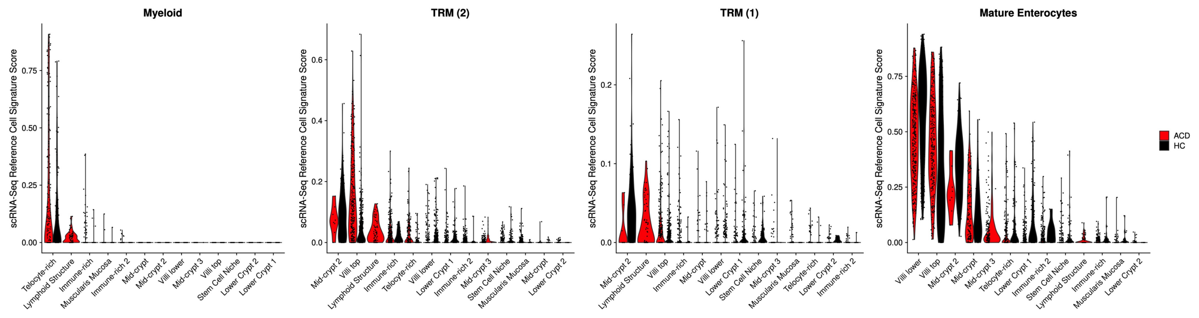

c

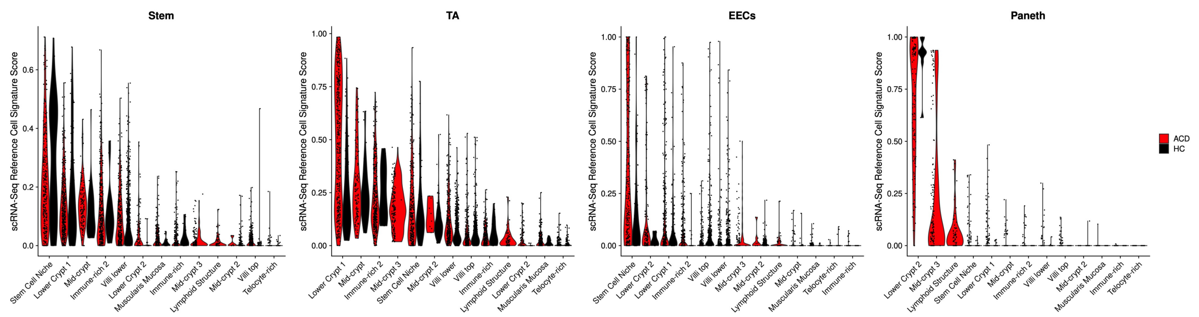

d

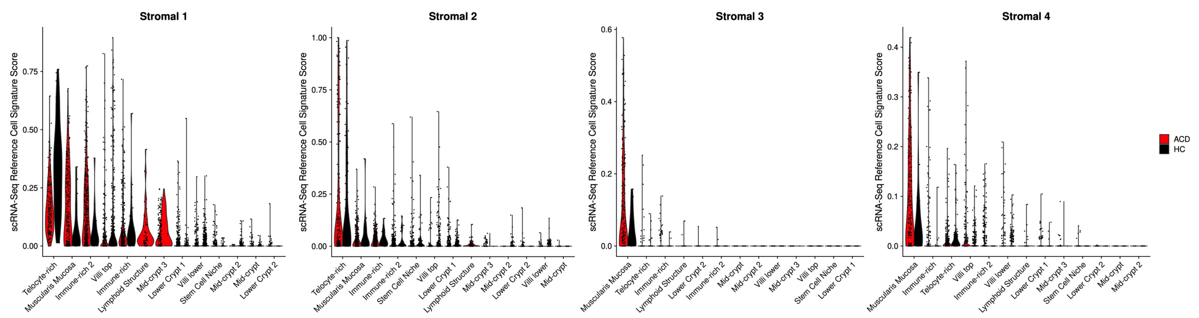

Supplementary Figure 7: Spatial Transcriptomics (2)

(a) Violin plots of Tfh-like $CD4^+$ cell, Treg cell, B cell and plasma cell specific transcriptional signatures in spatial regions, coloured by disease state. (b) Violin plots of myeloid cell, T_{RM}(2) $CD8^+$ T-cell, T_{RM}(1) $CD8^+$ T-cell, and mature enterocyte specific transcriptional signatures in spatial regions, coloured by disease state. (c) Violin plots of stem cell, transit-amplifying cell, enteroendocrine cell and Paneth-like cell specific transcriptional signatures in spatial regions, coloured by disease state. (d) Violin plots of stromal cell subtypes 1-4 transcriptional signatures in spatial regions, coloured by disease state. Stromal cell nomenclature from Kinchen et al. 2019.

Supplementary Figure 8

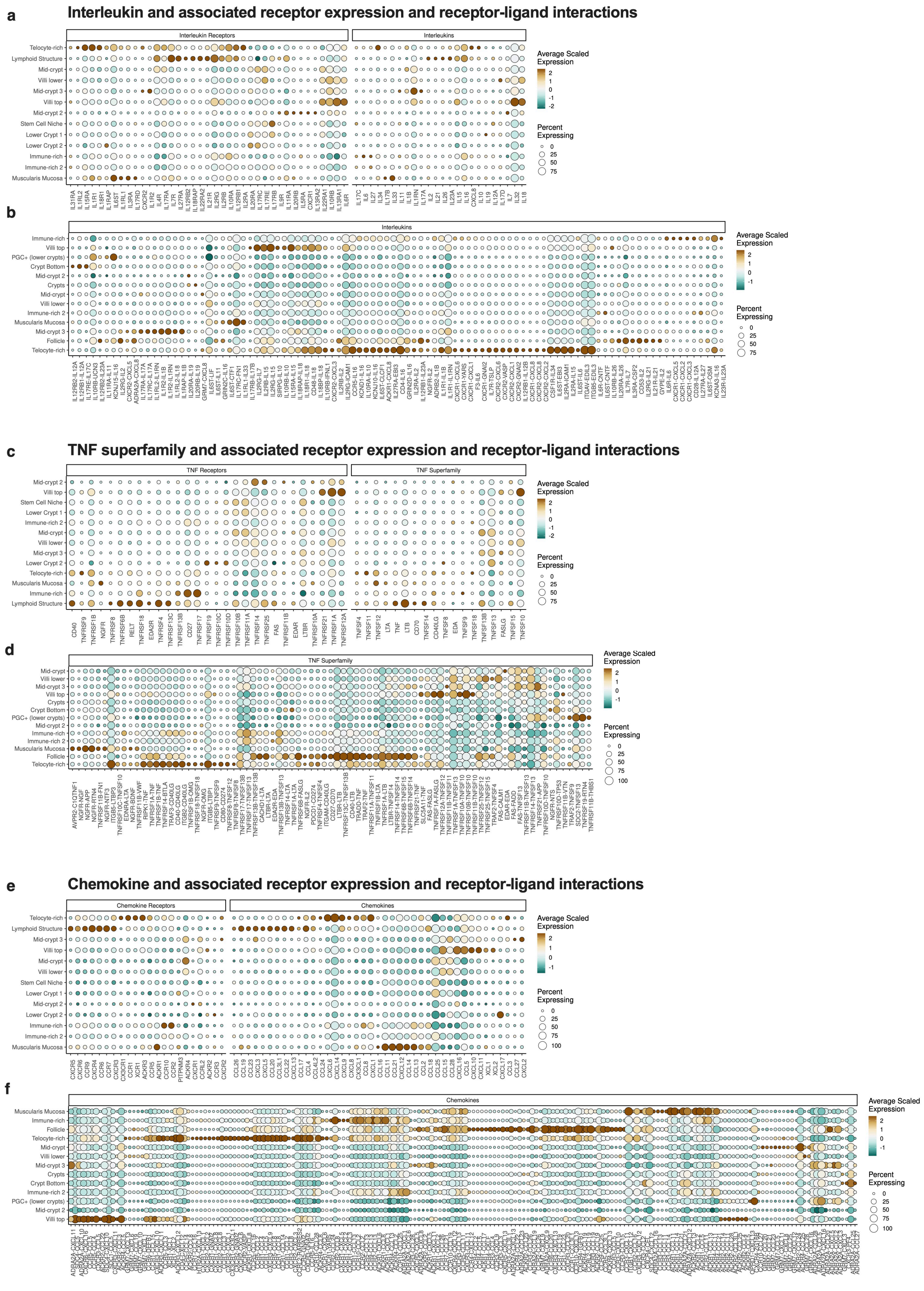

Supplementary Figure 8: Spatial Transcriptomics (3)

(a) Scaled expression of interleukins and associated receptors, and (b) interleukin receptor-ligand pair expression, in distinct spatial regions within duodenal mucosa. (c) Scaled expression of TNF superfamily members and associated receptors, and (d) TNF superfamily receptor-ligand pair expression, in distinct spatial regions within duodenal mucosa. (e) Scaled expression of chemokines and associated receptors, and (f) chemokine receptor-ligand pair expression, in distinct spatial regions within duodenal mucosa.

Description of Supplementary Tables (see .xlsx file)

1. Study participant demographics and clinical information
2. BD Rhapsody gene expression probes
3. BD Rhapsody AbSeq panel
4. Description of epithelial cell clusters
5. Differential expression of GO terms in epithelium (Active CD vs controls)
6. Description of CD4+ T-cell clusters
7. Description of CD8+ T-cell clusters

Supplementary Methods

Bulk TCR repertoire sequencing analysis

TCR repertoire library processing was performed using the iRepertoire analysis pipeline. In brief, reads were demultiplexed based on samples' 6-N molecular barcode. Low quality reads were trimmed (Phred score <30), and R1 and R2 reads were overlapped and stitched. Only stitched reads where identity within the overlapped portions was 100% were included in downstream analysis. Reads were mapped to the IMGT database, and only reads that map to reference sequences were included for further analysis. Finally, a number of filters (see irepertoire.com/irweb-technical-notes) were applied to remove sequencing artefacts, PCR artefacts, insertion, deletion, and substitution errors, and low frequency (n=1) reads.
Initial data analysis was performed using the iRweb data analysis platform (iRepertoire, Inc., USA). Additional analysis and generation of plots was performed using SeeTCR (friedmanlab.weizmann.ac.il/SeeTCR), Heatmaper[67] , Clustvis[68] .

Bulk RNA-seq analysis

Raw sequence reads were quality checked using FastQC. Cutadapt[69] was used to trim poor-quality bases (Phred Score <20) and Illumina universal adapter sequences from raw reads before alignment. The human hg38 reference genome analysis set was obtained from the University of California Santa Cruz (UCSC) ftp site[70] . The reference genome was indexed using STAR aligner[71] and sequenced reads were then aligned to this reference, using the following parameters:

```
STAR --runMode alignReads --genomeDir $genome --runThreadN 5 --readFilesIn $read1 $read2
\ --outFilterMismatchNoverLmax 0.1 --outFileNamePrefix $prefix --outSAMtype BAM
SortedByCoordinate \
--outFilterMultimapNmax 50 \
--outFilterMatchNminOverLread 0.75 \
--outFilterScoreMinOverLread 0.5 \
--outSAMattributes All \
--outFilterMatchNmin 10 \
--outFilterMismatchNmax 10 \
--outReadsUnmapped Fastx \
--readFilesCommand zcat \
```

Samtools[72] were used to merge, sort and index alignment bam files and to compute alignment statistics for alignment QC. Picard tools was used to mark duplicate sequences as an additional quality control step. Raw gene expression counts were summarized with featureCounts[73] , with multi-mapping reads counted as fractions of all mapped loci. The MultiQC tool[74] was used to aggregate quality metrics. Sample quality metrics and raw read counts were imported into R for further processing. The DESeq2[75] R package was used to estimate library size factors, normalize counts and perform differential expression analyses. Benjamini–Hochberg multiple testing correction was used to compute FDR, and genes were considered significantly differentially expressed at <5% FDR. Principal component analysis was performed in base R, with normalized DESeq2 variance-stabilized transformation expression as input. Correlation significance between principal components and variables of interest were computed in R using Kruskal-Wallis test for categorical variables and Spearman's correlation for continuous variables.

Raw single cell sequencing data processing

For all sequencing experiments, raw sequence reads were quality checked using FastQC software. For each sequenced 10x scRNA-Seq reaction, Cellranger software from 10 × Genomics (https://support.10xgenomics.com/single-cell-gene-expression/software/downloads/latest)
was used to process, align and summarize unique molecular identifier (UMI) counts against hg38 (10x reference: refdata-gex-GRCh38-2020-A) human reference genome. Paired single-cell TCR clonotypes were also assembled using Cellranger VDJ software. Spaceranger (version 1.2.2) was

used to process raw Visium spatial transcriptomics dataset, summarising counts against hg38 (10x reference: refdata-gex-GRCh38-2020-A), as before.

scRNA-Seq data analysis – 10x

Raw UMI count matrices were imported into R for further processing. For each scRNA-Seq sample, cell calling was performed using 'emptyDrops'[76] function from DropletUtils on the full raw count matrices of all barcodes in the 10x barcode whitelist to distinguish cells from empty droplets containing only ambient RNA. Raw count matrices were corrected for Illumina index swapping using 'swappedDrops'[77].

Furthermore, droplet barcodes for which a high percentage of total UMIs originated from mitochondrial RNAs were filtered out, as well as low total UMI count barcodes. These thresholds were derived individually for cells within each compartment following an initial clustering solution of all cells by examining and thresholding empirical distributions within each compartment, as total RNA content (notably higher in endothelial and myeloid cell populations) and mitochondrial RNA content (notably higher in epithelial cells) are highly cell type dependent. For each individual 10x reaction, Seurat R package[78] was used to normalize expression values for total UMI counts per cell. Highly variable genes were identified by fitting the mean-variance relationship and dimensionality reduction was performed using principal-component analysis. Scree plots were used to determine the number of principal components to use for clustering analyses for each pool. Cells were then clustered using Louvain algorithm for modularity optimization using kNN graph as input. Cell clusters were visualized using UMAP algorithm[79] with principal components as input and n.neighbors = 30, spread = 1 and min.dist = 0.1. Prior to final clustering analysis, cells from separate samples were merged together and batch effect signal was corrected using harmony algorithm[80], using default parameters.

scRNA-Seq cell populations were annotated using a combination of known marker gene and protein expression profiles, using previously published scRNA-Seq reference atlas datasets.

scRNA-Seq data analysis – BD Rhapsody

BD Rhapsody data were similarly processed using BD Genomics pipeline (version 1.9.1), using gencodev29-20181205 human reference genome build. Initially, read pairs with low quality were removed based on read length, mean base quality score and highest single-nucleotide frequency. The remaining high-quality R1 reads were analysed to identify cell label and unique molecular identifier (UMI) sequences. The remaining high-quality R2 reads were aligned to the reference panel sequences (mRNA and AbSeq) using Bowtie2. Reads with the same cell label, the same UMI sequence and the same gene were collapsed into a single molecule. The obtained counts were adjusted by BD Biosciences-developed error correction algorithms—recursive substitution error correction (RSEC) and distribution-based error correction (DBEC)—to correct sequencing and PCR errors. The DBEC-adjusted molecule counts obtained from the Rhapsody pipeline were imported, and the expression matrices were further analysed using the R package Seurat 3.0 & 4.3. Most cells identified as undetermined by the Rhapsody pipeline had a low number of features (mRNA and protein reads). These cells along with other cells with similarly low (< 35) number of features were filtered out. Identified multiplet cells were also filtered out at this stage. The resulting matrices were log normalised using the default parameters in Seurat, and the UMI counts were regressed out when scaling data. In this approach, protein (AbSeq) UMI counts were included in the same normalisation along with mRNA UMI counts.

Visium Spatial Transcriptomics Data Analysis

Raw UMI count spot matrices, images, spot-image coordinates and scale factors were imported into R. Spot matrix was filtered out to keep only spots overlaying tissue sections. We next fit a negative binomial distribution to total UMI counts in ST spots not under tissue sections to determine the expected recovery of UMI spots in non-tissue/technical background spots. We then additionally filtered out all under-tissue ST spots with low RNA content where total UMI recoveries were consistent with non-tissue spots, as these areas in tissue were likely under permeabilised. The majority of spots filtered out this way were either section-specific or

corresponded to tissue artefacts. Additional tissue artefacts (e.g., folds, tears) were further annotated in H&E and ST spots directly covering these regions were also excluded from further analysis.

Raw UMI spot counts were then normalized using regularised negative binomial regression (SC Transform)[81] to better account for variability in total spot RNA content. Dimensionality reduction was performed using PCA and for each slide, scree plots were examined the determine the optimum number of principal components to use in downstream clustering analyses. Clustering was performed using Louvain clustering algorithm as before (resolution = 0.5 (Dataset 4); resolution = 2 (Dataset5)) and clusters were visualized using UMAP algorithm as before. Clusters distributions were visualized in spatial context over H&E images with spot size scaling factor of 4.5.

For integrative data analysis, spots from individual slides were integrated using harmony algorithm correcting for slide-specific effects and clustering on merged dataset was carried out as before, except using harmony reduced dimension components instead of PCA. Merged data clusters were compared with those obtained from individual slides to ensure no heterogeneity was lost due to batch correction. Conversely, we examined individual slide contribution to integrated regions to ensure that equivalent regions between different tissue sections were clustering together. Clusters were annotated based on their corresponding anatomical region or in the case of disease-enriched clusters, dominant cellular composition.

Cell type prediction probabilities were calculated for each spot using factor analysis via FindTransferAnchors and TransferData functions in Seurat using scRNA-Seq reference dataset. We generated an integrated scRNA-Seq reference dataset covering all major cellular compartments by combining 10x scRNA-Seq dataset generated here.

For cell type co-occurrence analysis, in order to broadly assess the spatial co-localisation of cell populations within the same spots, we calculated all pairwise cell type prediction probability score correlations across all slides in disease and control conditions. To visualise cell type co-localisations in the three conditions, undirected, edge-weighted cell type networks were constructed from the correlation matrix from spots in each condition, retaining only significantly ($p < 0.01$) positively correlated cell type pair edges using R package 'igraph'[82], with correlation values as edge weights. Diagonals (cell type signal correlation to itself) were also filtered out. Due to the large number of cell types, for visualisation clarity we further filtered out edges below $r < 0.15$ to remove low correlation edges from the graphs across all conditions. Networks were visualised using R package 'ggraph', using force-directed Fruchterman-Reingold layout.

To identify region-specific spatially co-localised cellular signalling events, we first downloaded receptor-ligand databases[83,84] and scored all individual ST spots for receptor-ligand co-expression as follows. For each ST spot we also considered weighted receptor and ligand expression in surrounding spots. First, all pairwise Euclidean distances for all ST spots in all slides were computed and for each spot, a proximity-based linear weight was assigned to all other spots, with distal spots further than two immediately surrounding spots away assigned a weight of zero (no contribution towards co-localising receptor-ligand score) while other surrounding spots were assigned a distance-normalised weighting between >0 and 1. Then, for each spot a distance-weighted, local region smoothed receptor-ligand product score is calculated, which is further scaled to total non-zero weight spots to account of edge of tissue cases:

$$\frac{\sum_{i=1}^{n} d_i L_i}{n} \cdot \frac{\sum_{i=1}^{n} d_i R_i}{n}$$

Where L is ligand gene expression, R is receptor gene expression, d is distance-based weight and n is the number of spots with distance weight > 0. Next, we randomly shuffle all spot locations across all slides using 100 permutations and re-calculate the scores to compute an empirical background distribution that would be expected if there was no location specificity of receptor-ligand co-expression. Then for each spot and each receptor-ligand pair, we compute a p-value based on the empirical background distribution. A multiple testing Benjamini-Hochberg

correction is further applied to control false discovery rate as all receptor-ligand pairs are tested; spots with <5% FDR were then considered as positive for a cross-talk via a given receptor-ligand pair.
To prioritise region specific cross-talk events, we used generalised linear modelling, modelling receptor-ligand score dependence on each spatial region cluster, compared to spots in all other clusters and blocking for individual spot gene detection rate to account for variation in recoveries/permeabilization effectiveness between/within different slides. Similarly, condition-specific interactions were also modelled. We tested all receptor-ligand pairs which were detected as significantly co-localising in at least one spot in a tested region/condition. Benjamini-Hochberg multiple testing correction was further applied to control false discovery rate.
Spatial spot cluster adjacency networks were computed as follows. For each spot, we computed Euclidean distances to each other spot using the spot coordinates of ST images. Using distances, for any given spot, we selected immediately spatially adjacent spots (dist < 4 and dist > 0 with respect to the downscaled, low resolution image coordinates) and counted the fraction of the directly adjacent spots occupied by different crypt top region clusters. We then computed the mean surrounding spot cluster fractions for all "central" spots in each cluster and used these values as an edge weight to construct a weighted, directed network for visualising cluster spatial adjacency in disease and control slides. Diagonals (representing spots from the same transcriptome driven cluster occupying adjacent spots in tissue space) were kept. As before, R package 'igraph' was used to construct the network, 'ggpraph' was used to visualise the network. Networks were laid out for visualisation using a force-directed Fruchterman-Reingold layout.

Trajectory Analysis
Cell-differentiation trajectories were reconstructed using the R package monocle 2[85], in order to identify more simplified and interpretable trajectories than can be fit using more recent graph-based methods. Within each compartment, contaminating cells were filtered out prior to analysis (e.g. small number of immune contaminant cells within epithelial embedding). Dimensionality reduction was performed with the DDRTree algorithm, using all highly variable genes as inputs and technical co-variates/batches included in the residual model formula. Cell trajectory was then reconstructed using the orderCells function. In each case, starting node was denoted based on the most likely state based on prior biological knowledge – e.g. for epithelial cells, stem cells were used – and pseudotime was calculated from this node.

Transcription Factor Module Analysis
R package "SCENIC" workflow[86] was used to detect active transcription factor modules using transcriptional profiles of single cells from 10x data. Normalized single cell gene expression matrix was first filtered to exclude all genes detected in fewer than 20 total cells. The RcisTarget database containing transcription factor motif scores for gene promoters and around transcription start sites for hg38 human reference genome were downloaded from (https://resources.aertslab.org/cistarget/databases/homo_sapiens/hg38/refseq_r80/mc9nr/gene_based/) and the expression matrix was further filtered to only include genes available in the RcisTarget database. The remaining genes were used to compute a gene-gene correlation matrix for co-expression module detection using random forest based GENIE3 algorithm[87] and R package 'SCENIC' was used to perform transcription factor network analysis to detect co-expression modules enriched for target genes of each candidate TF from RcisTarget database. AUCell package was used to compute a score for each TF module in each individual cell as follows. First, for each cell single cell gene expression matrix was used to compute gene expression rankings using "AUCell_buildRankings" function with default parameters. Downstream targets of identified TF modules were assembled as gene sets and then used to score each cell, where for each gene set and each cell, area under the curve (AUC) value was computed ("AUCell_calcAUC" function) based on gene expression rankings, where AUC value represents the fraction of genes within the top ranking genes for each cell that are defined as part of the pathway gene set.
In order to identify differential TF module activities, we compared individual module AUC score distributions in single cells using generalised linear models. For each TF module, we fit a

generalised linear model with the formula of '*AUC ~ Condition + Gene Detection Rate +* Donor' to single cell data. Gene detection rate was included as an additional covariate, as we observed that AUC values are highly correlated individual cell library size. Condition coefficients and model p-values (corrected using Benjamini-Hochberg multiple testing correction) were then used to prioritise condition or cluster-specific enriched (positive coefficients) or underrepresented (negative coefficient) module TF module activities.
To visualise expression of all detected human TFs, we first downloaded a TF list from Lambert and colleagues[88] and filtered it to keep only genes annotated as TFs by consensus and at least minimally expressed within our 10x dataset. Expression patterns were visualised as mean, scaled expression level per cluster using hierarchical clustering analysis and heatmaps. To visualise distribution of expression patterns along pseudotime, we first divided all cells into 80 bins based on their position along pseudotime, as cell distribution along pseudotime is not uniform. Then, for each gene we fit a loess curve of pseudotime ~ expression and used the curve fits along pseudotime as input for hierarchical clustering of genes and visualisation via heatmaps.

γδ/CD8⁺ T cell classifier training (Dataset 8)

To discriminate γδ T cells from $CD8^+$ T cells, a random forest classifier was trained and validated using gene expression data. The model was trained using R randomForest (version 4.7-1.1) and caret (version 6.0-94) packages. "Ground truth" data from γδ T cells and $CD8^+$ T cells from FACS-sorted scRNA-Seq reactions were used for model training. First, the data was split into separate training and testing datasets, with a 2/3–1/3 ratio for model development and evaluation, respectively. Normalized gene expression values for all genes were used as input features. Feature selection was then performed to enhance model performance and interpretability, identifying the most informative genes based on their contribution to class separation. As expected, the selected features predominantly included γδ and αβ TCR chains, constant region genes and CD8A and CD8B gene expression. Using the identified features, the final model was then trained on the labelled dataset (ntree=300, all other parameters default), and applied to discriminate between γδ and $CD8^+$ T cells from unsorted pools.

Differential Gene Expression Analysis

Analyses of marker genes and differential gene expression across conditions in both Spatial Transcriptomics (ST) and single-cell RNA sequencing (scRNA-seq) data were conducted using a negative binomial generalized linear model, as implemented in Seurat wrapper. For each analysis, potential confounding factors such as variations in gene detection rates across spots or cells, as well as batch, donor, or slide effects, were accounted for in the statistical model as covariates for adjustment. The Benjamini-Hochberg method was applied for multiple testing correction to determine the false discovery rate (FDR). Genes that exhibited an FDR less than 5% were identified as significantly differentially expressed. Differential gene expression analyses for myeloid and stromal and myeloid datasets were performed using DESeq2 and visualised using the pheatmap and EnhancedVolcano R packages[89].

Differential Cell Type Abundance Analysis

To identify condition specific clusters, for each cluster for each sample within each cellular compartment we normalised cell counts to the total number of cells detected within that compartment in a given sample and the proportions of cells were compared using a two-sided Wilcoxon test, with p-values < 0.05 considered as significantly different. For cell type populations which exist on a phenotypic continuum rather than discrete clusters (e.g., T-cell subtypes), we further carried out graph-based differential abundance analysis using R package miloR[90], using integrated, batch-corrected harmony components for nearest neighbour graph reconstruction. We similarly tested for enrichment in Visium ST spots. For myeloid cells, differential abundance analysis was performed and visualised using the sccomp package in R[91].

Pathway Analysis

Analyses to identify enriched Gene Ontology terms and pathways in the results obtained from bulk, scRNA-seq, and spatial transcriptomics datasets were conducted using the clusterProfiler package in R[92]. To map gene identifiers, the annotation package org.Hs.eg.db from Dbi R was utilized. Tests for overrepresentation were performed separately for sets of cluster markers and differentially expressed genes, using the list of all expressed or detected genes in each dataset as a reference background. The hypergeometric test P values were adjusted for multiple testing using the Benjamini–Hochberg method, as in previous analyses. The findings were then visualized employing the R packages clusterProfiler and ggplot2.

VDJ Analysis
Single-cell TCR clonotypes were assembled using Cellranger VDJ software. Single-cell barcodes were then used to link corresponding VDJ clonotypes and gene expression data. To examine overlaps of TCR repertoires between populations and assess phenotypic conversion and trafficking, we computed Morisita's index using R divo package between all pairwise clusters. UpsetR R package[93] was used to visualise cluster shared clonotype intersections.

Gene Family Analysis
Gene sets for immune-related gene family analyses (e.g. chemokines, interleukins) were download from HGNC database[94]. Genes which were not expressed/detected in any cells in the dataset were excluded from visualisations.

GWAS Enrichment
GWAS gene expression enrichment in specific single cell clusters was tested using SNPsea algorithm[95]. GWAS gene set was obtained from GWAS catalog[96]. A 'pseudobulk' dataset for each previously identified cell cluster was created from scRNA-Seq data by summing all UMI counts for each gene in each cluster. We then normalized the data by computing size factors using R package DESeq2 to account for differences in cell-cluster sizes.

Supplementary Methods References

67. Babicki, S. *et al.* Heatmapper: web-enabled heat mapping for all. *Nucleic Acids Res.* **44**, W147–W153 (2016).
68. Metsalu, T. & Vilo, J. ClustVis: a web tool for visualizing clustering of multivariate data using Principal Component Analysis and heatmap. *Nucleic Acids Res.* **43**, W566–W570 (2015).
69. Martin, M. Cutadapt removes adapter sequences from high-throughput sequencing reads. *EMBnetJ.* **17**, 10–12 (2011).
70. Kuhn, R. M., Haussler, D. & Kent, W. J. The UCSC genome browser and associated tools. *Briefings in Bioinformatics* **14**, 144–161 (2013).
71. Dobin, A. *et al.* STAR: ultrafast universal RNA-seq aligner. *Bioinformatics* **29**, 15–21 (2012).
72. Li, H. *et al.* The Sequence Alignment/Map format and SAMtools. *Bioinformatics* **25**, 2078–2079 (2009).
73. Liao, Y. *et al.* featureCounts: an efficient general purpose program for assigning sequence reads to genomic features. *Bioinformatics* **30**, 923–930 (2014).
74. Ewels, P., Magnusson, M., Lundin, S. & Käller, M. MultiQC: summarize analysis results for multiple tools and samples in a single report. *Bioinformatics* **32**, 3047–3048 (2016).
75. Love, M. I., Huber, W. & Anders, S. Moderated estimation of fold change and dispersion for RNA-seq data with DESeq2. *Genome biology* **15**, 31–21 (2014).
76. Lun, A. T. L. *et al.* EmptyDrops: distinguishing cells from empty droplets in droplet-based single-cell RNA sequencing data. *Genome Biol.* **20**, 63 (2019).
77. Griffiths, J. A., Richard, A. C., Bach, K., Lun, A. T. L. & Marioni, J. C. Detection and removal of barcode swapping in single-cell RNA-seq data. *Nat. Commun.* **9**, 2667 (2018).

78. Butler, A., Hoffman, P., Smibert, P., Papalexi, E. & Satija, R. Integrating single-cell transcriptomic data across different conditions, technologies, and species. *Nature Biotechnology* **36**, 411–420 (2018).
79. McInnes, L., Healy, J. & Melville, J. UMAP: Uniform Manifold Approximation and Projection for Dimension Reduction. *arXiv* (2018) doi:10.48550/arxiv.1802.03426.
80. Korsunsky, I. *et al.* Fast, sensitive and accurate integration of single-cell data with Harmony. *Nat. Methods* **16**, 1289–1296 (2019).
81. Hafemeister, C. & Satija, R. Normalization and variance stabilization of single-cell RNA-seq data using regularized negative binomial regression. *Genome Biol.* **20**, 296 (2019).
82. Csardi & Nepusz, &. The Igraph Software Package for Complex Network Research. *InterJournal, Complex Systems* **1695**, 1–9 (2006).
83. Cabello-Aguilar, S. *et al.* SingleCellSignalR: inference of intercellular networks from single-cell transcriptomics. *Nucleic Acids Res.* **48**, e55–e55 (2020).
84. Jin, S. *et al.* Inference and analysis of cell-cell communication using CellChat. *Nat. Commun.* **12**, 1088 (2021).
85. Qiu, X. *et al.* Reversed graph embedding resolves complex single-cell trajectories. *Nat. Methods* **14**, 979–982 (2017).
86. Aibar, S. *et al.* SCENIC: single-cell regulatory network inference and clustering. *Nat. Methods* **14**, 1083–1086 (2017).
87. Huynh-Thu, V. A., Irrthum, A., Wehenkel, L. & Geurts, P. Inferring Regulatory Networks from Expression Data Using Tree-Based Methods. *PLoS ONE* **5**, e12776-10 (2010).
88. Lambert, S. A. *et al.* The Human Transcription Factors. *Cell* **172**, 650–665 (2018).
89. Blighe, K., Rana, S. & M, L. EnhancedVolcano: Publication-ready volcano plots with enhanced colouring and labeling. R package version 1.24.0. https://github.com/kevinblighe/EnhancedVolcano. (2024).
90. Dann, E., Henderson, N. C., Teichmann, S. A., Morgan, M. D. & Marioni, J. C. Differential abundance testing on single-cell data using k-nearest neighbor graphs. *Nat. Biotechnol.* **40**, 245–253 (2022).
91. Mangiola, S. *et al.* sccomp: Robust differential composition and variability analysis for single-cell data. *Proc. Natl. Acad. Sci.* **120**, e2203828120 (2023).
92. Yu, G., Wang, L.-G., Han, Y. & He, Q.-Y. clusterProfiler: an R Package for Comparing Biological Themes Among Gene Clusters. *OMICS: A Journal of Integrative Biology* **16**, 284–287 (2012).
93. Conway, J. R., Lex, A. & Gehlenborg, N. UpSetR: an R package for the visualization of intersecting sets and their properties. *Bioinformatics* **33**, 2938–2940 (2017).
94. Seal, R. L. *et al.* Genenames.org: the HGNC resources in 2023. *Nucleic Acids Res.* **51**, D1003–D1009 (2022).
95. Slowikowski, K., Hu, X. & Raychaudhuri, S. SNPsea: an algorithm to identify cell types, tissues and pathways affected by risk loci. *Bioinformatics* **30**, 2496–2497 (2014).
96. MacArthur, J. *et al.* The new NHGRI-EBI Catalog of published genome-wide association studies (GWAS Catalog). *Nucleic Acids Res.* **45**, D896–D901 (2017).
