# Supplementary material for: Immune–epithelial–stromal networks define the cellular ecosystem of the small intestine in celiac disease
Source: Nat Immunol. 2025 May 6;26(6):947–62. doi: 10.1038/s41590-025-02146-2 (PMC12133578; doi:10.1038/s41590-025-02146-2)
Supplement: Supplementary file 2 — Reporting Summary [file 41590_2025_2146_MOESM2_ESM.pdf]

Corresponding author(s): Michael FitzPatrick

Last updated by author(s): 20-Mar-2025

Reporting Summary

Nature Portfolio wishes to improve the reproducibility of the work that we publish. This form provides structure for consistency and transparency in reporting. For further information on Nature Portfolio policies, see our Editorial Policies and the Editorial Policy Checklist.

Statistics

For all statistical analyses, confirm that the following items are present in the figure legend, table legend, main text, or Methods section.

n/a	Confirmed	
☐	☒	The exact sample size (*n*) for each experimental group/condition, given as a discrete number and unit of measurement
☐	☒	A statement on whether measurements were taken from distinct samples or whether the same sample was measured repeatedly
☐	☒	The statistical test(s) used AND whether they are one- or two-sided<br>*Only common tests should be described solely by name; describe more complex techniques in the Methods section.*
☐	☒	A description of all covariates tested
☐	☒	A description of any assumptions or corrections, such as tests of normality and adjustment for multiple comparisons
☐	☒	A full description of the statistical parameters including central tendency (e.g. means) or other basic estimates (e.g. regression coefficient) AND variation (e.g. standard deviation) or associated estimates of uncertainty (e.g. confidence intervals)
☐	☒	For null hypothesis testing, the test statistic (e.g. *F*, *t*, *r*) with confidence intervals, effect sizes, degrees of freedom and *P* value noted<br>*Give P values as exact values whenever suitable.*
☐	☒	For Bayesian analysis, information on the choice of priors and Markov chain Monte Carlo settings
☐	☒	For hierarchical and complex designs, identification of the appropriate level for tests and full reporting of outcomes
☐	☒	Estimates of effect sizes (e.g. Cohen's *d*, Pearson's *r*), indicating how they were calculated

Our web collection on statistics for biologists contains articles on many of the points above.

Software and code

Policy information about availability of computer code

Data collection	No software used for data collection
Data analysis	FlowJo v9.9.5 and v10.6.1 (FlowJo LLC).<br>Prism Software v9 & v10 (GraphPad, USA).<br>R studio Version 2023.06.0.<br>All software and code used in R are listed in Supplementary Methods.

For manuscripts utilizing custom algorithms or software that are central to the research but not yet described in published literature, software must be made available to editors and reviewers. We strongly encourage code deposition in a community repository (e.g. GitHub). See the Nature Portfolio guidelines for submitting code & software for further information.

Data

Policy information about availability of data

All manuscripts must include a data availability statement. This statement should provide the following information, where applicable:

- Accession codes, unique identifiers, or web links for publicly available datasets
- A description of any restrictions on data availability
- For clinical datasets or third party data, please ensure that the statement adheres to our policy

Raw and processed data are available via Zenodo (https://doi.org/10.5281/zenodo.15069144). Genomic data is also deposited at GSE252545. Re-analysed data from Martin et al. at GSE134809.

Research involving human participants, their data, or biological material

Policy information about studies with human participants or human data. See also policy information about sex, gender (identity/presentation), and sexual orientation and race, ethnicity and racism.

Reporting on sex and gender	Data on sex is reported in Supplementary Table 1
Reporting on race, ethnicity, or other socially relevant groupings	No reporting on race or ethnicity is included
Population characteristics	Demographic data and data on disease state is included in Supplementary Table 1
Recruitment	Study subjects with CD, and healthy controls, were identified via Oxford University Hospitals NHS Trust (OUH) celiac disease clinic and endoscopy service (Oxford, UK). Blood and intestinal biopsies were taken at endoscopy with informed under the Oxford Gastrointestinal Illnesses Biobank study (REC: 21/YH/0206). Study subject demographics and study inclusion/exclusion criteria, are summarised in Supplementary Table 1. Participants were not compensated financially. No potential biases in recruitment are identified.
Ethics oversight	Study sponsor - University of Oxford. Study ethical approval: Oxford Gastrointestinal Illnesses Biobank study (REC: 21/YH/0206) - Yorkshire & The Humber - Sheffield Research Ethics Committee.

Note that full information on the approval of the study protocol must also be provided in the manuscript.

Field-specific reporting

Please select the one below that is the best fit for your research. If you are not sure, read the appropriate sections before making your selection.

☒ Life sciences ☐ Behavioural & social sciences ☐ Ecological, evolutionary & environmental sciences

For a reference copy of the document with all sections, see nature.com/documents/nr-reporting-summary-flat.pdf

Life sciences study design

All studies must disclose on these points even when the disclosure is negative.

Sample size	This was an observational, descriptive study. No statistical method was used to predetermine sample size, but our sample sizes are similar to those reported in previous publications(Ref 28,32,36).
Data exclusions	No data were excluded from analyses.
Replication	Replication of scRNA-seq experiments in two cohorts. Replication of spatial transcriptomics in two cohorts. Replication of TCR-seq findings in several experiments.
Randomization	The experiments were not randomised as this was an observational study.
Blinding	Data collection and analysis were not performed blind to the conditions of the experiments.

Reporting for specific materials, systems and methods

We require information from authors about some types of materials, experimental systems and methods used in many studies. Here, indicate whether each material, system or method listed is relevant to your study. If you are not sure if a list item applies to your research, read the appropriate section before selecting a response.

Materials & experimental systems

n/a	Involved in the study
	☒ Antibodies
☒	Eukaryotic cell lines
☒	Palaeontology and archaeology
☒	Animals and other organisms
☒	Clinical data
☒	Dual use research of concern
☒	Plants

Methods

n/a	Involved in the study
☒	ChIP-seq
	☒ Flow cytometry
☒	MRI-based neuroimaging

Antibodies

Antibodies used: Antibodies used in AbSeq experiments in Supplementary Table 3.

Validation: Full details of all antibodies listed in Supplementary Table 3 can be found on the supplier's website: https://www.bdbiosciences.com/en-eu/products/reagents/single-cell-multiomics/abseq

Plants

Seed stocks: N/A

Novel plant genotypes: N/A

Authentication: N/A

Flow Cytometry

Plots

Confirm that:

☒ The axis labels state the marker and fluorochrome used (e.g. CD4-FITC).

☒ The axis scales are clearly visible. Include numbers along axes only for bottom left plot of group (a 'group' is an analysis of identical markers).

☒ All plots are contour plots with outliers or pseudocolor plots.

☒ A numerical value for number of cells or percentage (with statistics) is provided.

Methodology

Sample preparation: For surface marker staining, cells were stained in 50 mL of FACS buffer (PBS + 1mM EDTA + 0.05% BSA) for 30 minutes at 4 C. Surface antibodies and clones used are listed in Supplementary Table 3. Antibodies were purchased from BioLegend, BD Biosciences, Miltenyi Biotec, or Thermo Fisher Scientific.
After staining, cells were stored at 4 C protected from light until data acquisition. For fluorescence-activated cell sorting (FACS) samples were surface stained as above, with Sytox Green (Thermo Fisher Scientific) used as viability dye.
For sorting by FACS for scRNA-seq of intestinal immune populations, cells were stained with EpCam-PE, CD27-BV421, and CD45-APC-Cy7. Live CD45+ or CD27+ cells were sorted to include all mucosal immune cell populations, including long-lived CD27+ plasma cells which can downregulate surface CD45 expression.
For sorting by FACS for scRNA-seq of intestinal epithelial populations, cells were stained with EpCAM-PE and CD45-AF700, with live EpCAM+ cells sorted.
For sorting by FACS for bulk RNA-seq or TCR-seq of CD8+ intraepithelial lymphocyte populations, cells were stained with CD45-BV785, CD3-BV711, αβTCR-APC, γδTCR-PE, CD4-BV650, CD8a-AF700, with live, CD45+, CD3+, αβTCR+, CD8+, CD4- cells sorted.

Instrument: Flow cytometry data were acquired on a BD LSRII flow cytometer (BD Biosciences).FACS was performed on an AriaIII (BD Biosciences; 70 mm nozzle).

Software: FlowJo v9.9.5 and v10.6.1 (FlowJo LLC).

Cell population abundance	Post-sort purity was confirmed with purity > 98%.
Gating strategy	For sorting by FACS for scRNA-seq of intestinal immune populations, cells were stained with EpCam-PE, CD27-BV421, and CD45-APC-Cy7. Live CD45+ or CD27+ cells were sorted to include all mucosal immune cell populations, including long-lived CD27+ plasma cells which can downregulate surface CD45 expression.<br>For sorting by FACS for scRNA-seq of intestinal epithelial populations, cells were stained with EpCAM-PE and CD45-AF700, with live EpCAM+ cells sorted.<br>For sorting by FACS for bulk RNA-seq or TCR-seq of CD8+ intraepithelial lymphocyte populations, cells were stained with CD45-BV785, CD3-BV711, αβTCR-APC, γδTCR-PE, CD4-BV650, CD8a-AF700, with live, CD45+, CD3+, αβTCR+, CD8+, CD4- cells sorted.<br>Flow cytometry gating strategy is shown in Ext. Fig. 7.

☒ Tick this box to confirm that a figure exemplifying the gating strategy is provided in the Supplementary Information.
